# Interaction of human hemoglobin and semi-hemoglobins with the *Staphylococcus aureus* hemophore IsdB: a kinetic and mechanistic insight

**DOI:** 10.1038/s41598-019-54970-w

**Published:** 2019-12-09

**Authors:** Eleonora Gianquinto, Ilaria Moscetti, Omar De Bei, Barbara Campanini, Marialaura Marchetti, F. Javier Luque, Salvatore Cannistraro, Luca Ronda, Anna Rita Bizzarri, Francesca Spyrakis, Stefano Bettati

**Affiliations:** 1 Department of Drug Science and Technology, University of Turin, Turin, 10125 Italy; 2 Department of Environmental and Biological Sciences, University of Tuscia, Viterbo, 01100 Italy; 3 Department of Food and Drug, University of Parma, Parma, 43124 Italy; 4 Interdepartment Center Biopharmanet-TEC, University of Parma, Parma, 43124 Italy; 5 Department of Nutrition, Food Science and Gastronomy, Faculty of Pharmacy and Food Sciences, Institute of Biomedicine (IBUB) and Institute of Theoretical and Computational Chemistry (IQTCUB), University of Barcelona, Santa Coloma de Gramenet, 08921 Spain; 6 Department of Medicine and Surgery, University of Parma, Parma, 43126 Italy; 7 Institute of Biophysics, National Research Council, Pisa, 56124 Italy

**Keywords:** Biophysics, Computational biology and bioinformatics

## Abstract

Among multidrug-resistant bacteria, methicillin-resistant *Staphylococcus aureus* is emerging as one of the most threatening pathogens. *S. aureus* exploits different mechanisms for its iron supply, but the preferred one is acquisition of organic iron through the expression of hemoglobin (Hb) receptors. One of these, IsdB, belonging to the Isd (Iron-Regulated Surface Determinant) system, was shown to be essential for bacterial growth and virulence. Therefore, interaction of IsdB with Hb represents a promising target for the rational design of a new class of antibacterial molecules. However, despite recent investigations, many structural and mechanistic details of complex formation and heme extraction process are still elusive. By combining site-directed mutagenesis, absorption spectroscopy, surface plasmon resonance and molecular dynamics simulations, we tackled most of the so far unanswered questions: (i) the exact complex stoichiometry, (ii) the microscopic kinetic rates of complex formation, (iii) the IsdB selectivity for binding to, and extracting heme from, α and β subunits of Hb, iv) the role of specific amino acid residues and structural regions in driving complex formation and heme transfer, and (v) the structural/dynamic effect played by the hemophore on Hb.

## Introduction

The ability of bacterial pathogens to establish infections relies on the adaptation of bacterial metabolism to the environment found within the host, which is often severely nutrient-restricted and a site for competition with commensal microorganisms and other pathogens^1,2^. A well-characterized mechanism of adaptation to the nutritional environment of the host is represented by the expression of redundant iron-acquisition systems, both in Gram^+^ and Gram^-^ bacteria, to provide iron supply to support invasion and proliferation^3^. Iron is an essential nutrient for both the pathogen and the host, but also a toxic element due to its involvement in Fenton chemistry and Haber-Weiss reactions that generate reactive oxygen species (ROS)^4^. For this reason, vertebrates have put in place strategies to maintain very low concentrations of free iron in body fluids (down to 10^−18^–10^−24^ M)^4,5^ achieving two goals, the first being limiting toxicity and the second creating an iron-restricted environment for pathogens^6,7^. In *S. aureus*, iron scavenging is achieved by at least three different mechanisms: i) acquisition of inorganic iron through production of siderophores, ii) expression of hemoglobin (Hb) receptors, and iii) acquisition of inorganic iron through transporters^4^. Heme is the preferred iron source during the initial phase of infection^8^ and, indeed, *S. aureus* expresses two Hb receptor isoforms, called IsdB^5^ and IsdH^9–11^. They both belong to the so-called Isd (Iron-regulated Surface Determinant) system that comprises nine proteins (IsdA-I) responsible for heme extraction from Hb, heme transport through the cell wall and the membrane, and heme disassembly to get free ferrous ions. IsdB is responsible for heme acquisition from Hb only, whereas IsdH can capture heme from both free Hb and Hb-haptoglobin complex^12,13^. IsdB is strongly up-regulated under conditions of iron restriction^14^. Different experimental evidences pointed out that IsdB is crucial for *S. aureus* proliferation and virulence. Δ*isdB* strains or strains expressing Hb-binding deficient mutants show severe growth defects in media with Hb as the sole iron source and have a reduced colonization capacity^15^. More recently the role of IsdB in adhesion to host cells has been pointed out, further complicating the description of its function in host-pathogen interaction and in virulence^16,17^. Its role as a virulence factor has been also recently assessed by analyzing 30 West Australian methicillin-resistant *S. aureus* (MRSA) isolates of human origin, where the IsdB gene was identified in the vast majority of cases^18^.

**Figure 1:**
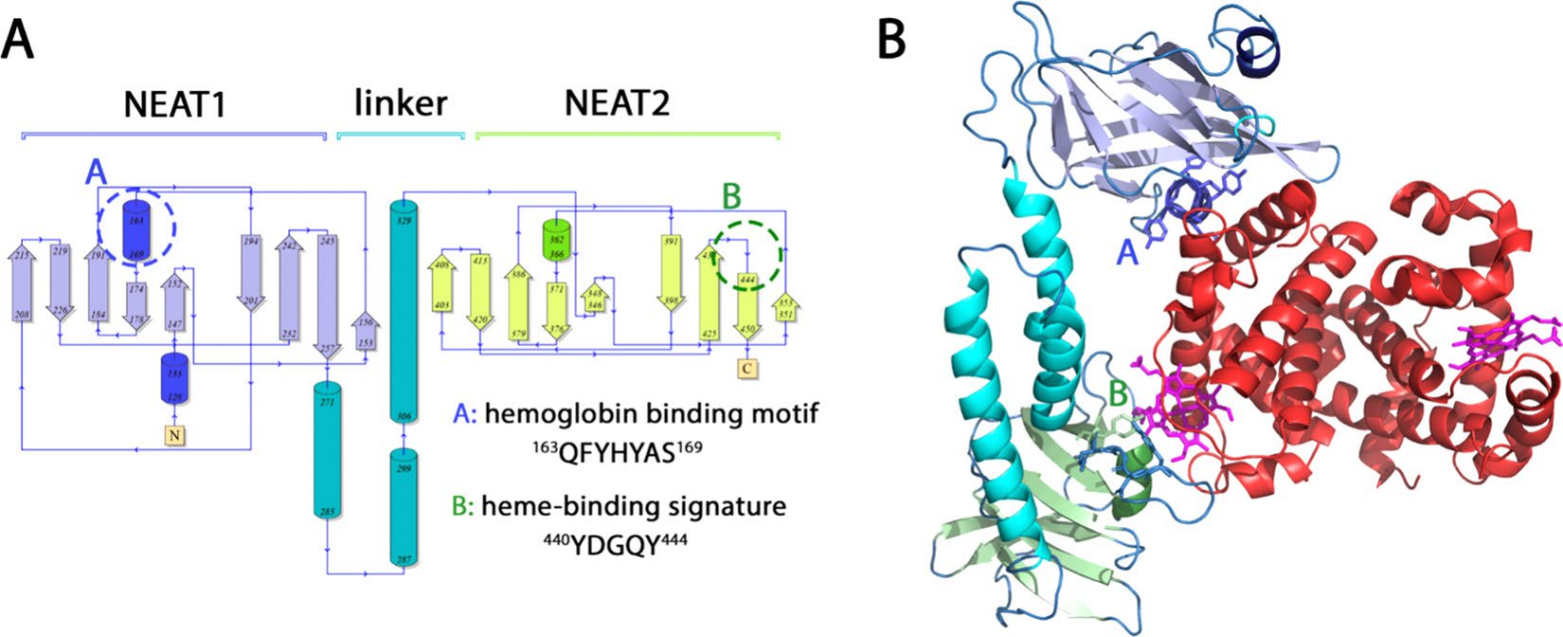
IsdB secondary and tertiary structures. (**A**) Topology of IsdB (PDB code 5vmm). The structure consists of 18 β-strands and 6 α-helices, forming two NEAT domains connected by a flexible linker. (**B**) 3D cartoon structure of wt IsdB in complex with methemoglobin (MetHb). Hb binding motif and heme binding signature are represented in stick residues. A and B refer, respectively, to the hemoglobin binding motif and to the heme-binding signature.

IsdB (Fig. 1) is characterized by a modular structure formed by the assembly of two NEAT domains (referred to as N1 and N2). NEAT domains are heme/Hb binding modules conserved in Gram^+^ bacteria. They were firstly identified by a bioinformatic search of putative iron transporters genic loci (the acronym stands for NEAr-iron Transporter^19^). NEAT domains display a β-sandwich fold with a central 3_10_-helix that forms a hydrophobic cavity where the heme is eventually bound^20^. The NEAT domains are linked by rigid α-helical bundles which contribute to high affinity binding of IsdB to Hb, as suggested by pull-down assays^20^. NEAT domains can perform three actions: direct heme binding, Hb binding and heme extraction, and heme transfer between NEAT domains of separate Isd receptors. The “heme-binding signature”^21^ of *S. aureus* and *Bacillus anthracis* NEAT domains is the five-residues sequence YXXXY, where the first Tyr residue directly binds the heme iron and the second is hydrogen-bonded to the first one^22^. Interestingly, IsdB uses only one out of two NEAT domains to bind the heme. Indeed, the two NEAT domains of IsdB have very low sequence identity (about 11%) and the C-terminal one (N2) carries the heme-binding signature sequence YDGQY, which is absent in the N-terminal sequence (N1). The N1 domain exhibits the FYHYA sequence that is highly conserved in different *S. aureus* strains and is essential for Hb binding, as recently confirmed by Ala-scanning experiments^15^. Therefore, the N1 domain of IsdB is involved in Hb binding while N2 is involved in heme extraction^23^.

As also recently pointed out for IsdH^24^, although several studies have undertaken the structural and functional characterization of the IsdB/Hb complex^13,20,25^, the complicated nature of the protein-protein interaction has often prevented to obtain concluding results about the complex dynamics^24^. Six 3D structures of IsdB, and about the double for IsdH, have been deposited in the Protein Data Bank up to now. None of them reports the full-length structure of the hemophores and only fragments comprising one or more domains are present. On the other hand, seven structures of IsdH/IsdB in complex with Hb have been deposited, with only three including more than one NEAT domain. The first two structures of methemoglobin (MetHb) in complex with IsdH N2 and N3 were solved by Dickson and co-workers in 2014 and 2015^26,27^. Differently from IsdB, in fact, IsdH presents three NEAT domains, of which N1 and N2 are involved in binding Hb and N3 in extracting the heme.

The unique structure of the IsdB/Hb complex, solved by Bowden *et al*. in 2017, presents low resolution, heme dislocation and Hb helix F unfolding^13^. Any improvement in the knowledge of this fascinating, although complicated, system is crucial to better understand the dynamics of similar systems in different pathogens and, eventually, to envisage innovative antimicrobial approaches. In this regard, the main still open questions involve (i) the exact complex stoichiometry in solution^27^; (ii) the microscopic kinetic rates that drive complex formation; (iii) the existence of a different efficiency for heme extraction from α and β subunits of Hb, still strongly controversial^13,28^; (iv) the role of specific amino acid residues in driving complex formation and heme transfer, and (v) the structural/dynamic effect played by the hemophores on Hb.

We have addressed all these points by a combined *in vitro* and *in silico* approach. We investigated by UV-Vis spectroscopy and by surface plasmon resonance (SPR) the interaction of wild-type IsdB and the Y440A variant (IsdB^Y440A^, expected to be unable to complete heme transfer^13,22^), with human Hb and with semiHbs. SemiHbs are stable Hb α/β dimers where either the α (α-semiHb) or the β (β-semiHb) subunits bind heme^29^ and are here used to detect any subunit preference in IsdB binding and heme extraction. *In silico* simulations were run to investigate the IsdB/Hb interface and identify the key residues involved in the complex recognition and in the heme extraction. Essential dynamics analyses helped us to investigate the dynamics of the complex and the structural changes induced on Hb by hemophore binding.

## Results

### Protein characterization

**Figure 2:**
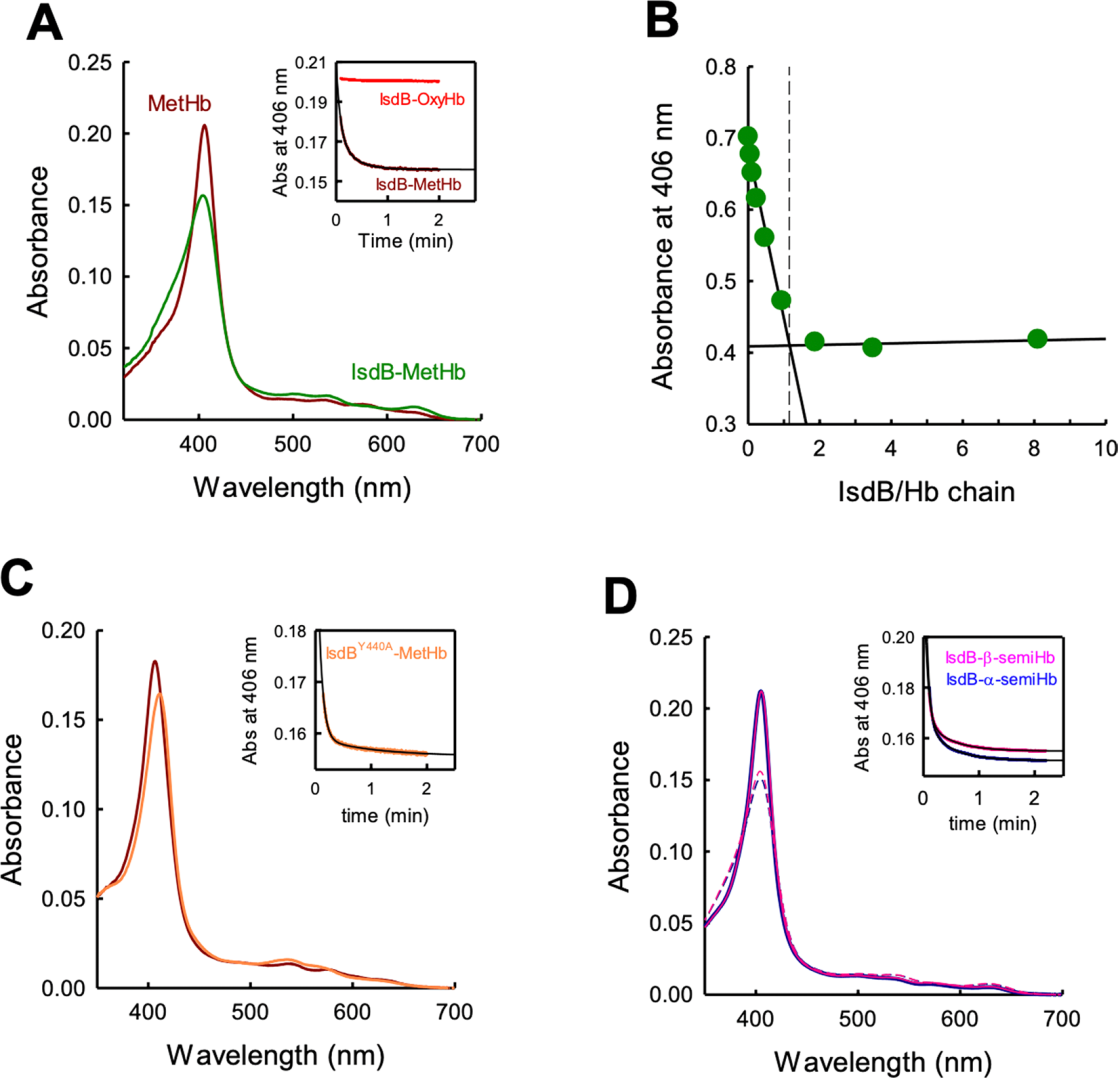
Spectroscopic characterization of heme extraction from Hb and semiHb by wt IsdB and IsdB^Y440A^. (**A**) Absorption spectra of 1.5 µM MetHb before (brown) and after (green) mixing with 7.5 µM IsdB. Inset: time-dependence of absorbance at 406 nm upon mixing IsdB with OxyHb (red) or MetHb (brown). (**B**) Stoichiometric titration of MetHb with IsdB. (**C**) Absorption spectra of 1.5 µM MetHb before (brown) and after (orange) mixing with 7.5 µM IsdB^Y440A^. Inset: time-dependence of absorbance at 406 nm upon mixing IsdB^Y440A^ with MetHb. (**D**) Absorption spectra of 1.5 µM α-semiHb (dark blue) or β-semiHb (pink) before (continuous line) and after (dashed line) mixing with 7.5 µM IsdB. Inset: time-dependence of absorbance at 406 nm upon mixing IsdB with α-semiHb (dark blue) or β-semiHb (pink). Black lines through kinetic traces are the fitting to a biexponential equation with parameters reported in Table 1.

IsdB from *S. aureus* used in this study was designed to contain the two NEAT domains (N1 and N2) connected by the helical linker, and a C-terminal Strep-Tag^®^ for protein purification and immobilization on SPR sensor chips. The wild-type protein and its variant IsdB^Y440A^ were purified in high yields, about 100 mg/L bacterial culture, and good homogeneity (Fig. S1A,B, respectively). The extinction coefficient at 280 nm is 47,800 M^−1^ cm^−1^ and 46,300 M^−1^ cm^−1^ for IsdB and IsdB^Y440A^, respectively. The extinction coefficient at 405 nm is 90,500 M^−1^ cm^−1^ and was calculated by using the pyridine hemochromagen assay^30^. The amount of holoIsdB was calculated to be less than 5% in the final preparation (Fig. S1C) and thus its contribution to Hb binding in the following experiments can be considered negligible. When IsdB is added to a solution of reduced, oxygenated Hb (OxyHb), the spectrum does not change and the absorbance at 406 nm is stable over time (Fig. 2A, inset). However, when IsdB is added to a MetHb solution (likely the dominant form interacting with the hemophore under *in vivo* infection conditions), a fast change in the absorbance spectrum is observed, with a decrease in the absorption maximum and a small blue-shift (Fig. 2A). This result is in agreement with literature data^15,20,25,31^ and is the spectroscopic signature of the heme transfer from Hb to IsdB. The transfer is completed in about 1 minute at 20 °C under pseudo-first order conditions, *i.e*. 7.5 µM IsdB and 1.5 µM Hb (Fig. 2A, inset). Fitting of the kinetic trace with a biexponential equation gives an observed kinetic rate k_obs_ for the fast phase (attributed to heme transfer) of 0.2 s^−1^ (Table 1), in very good agreement with published data collected under similar conditions^13,24^. The spectroscopic changes associated to the heme transfer were used to perform a stoichiometric titration of Hb binding to IsdB, since the stoichiometries of the IsdB/Hb complex reported in literature are not fully consistent^20^. When increasing IsdB concentrations are added to a MetHb solution the signal at 406 nm progressively decreases until the stoichiometric ratio is reached. Further addition of IsdB does not change the absorbance signal (Fig. 2B). The calculated stoichiometric ratio is 1, meaning that each Hb monomer binds one IsdB molecule. The IsdB^Y440A^ mutant elicits a red shift in the absorption spectrum of heme, suggesting that its final microenvironment is different from that experienced with wt IsdB. The final spectrum is centered at 410 nm (Fig. 2C). When IsdB is added to a solution containing either α-semiHb or β-semiHb, spectroscopic changes similar to those registered with native Hb are observed (Fig. 2D). Of notice, the rates of heme transfer, under identical conditions as those used for native Hb (Fig. 2D, inset), are very similar (k_obs_ of 0.28 s^−1^ and 0.27 s^−1^, respectively, for the fast kinetic phase), suggesting that IsdB can extract the heme with comparable efficiency from α and β subunits (vide infra).

**Table 1:** Observed rate constants and relative amplitudes obtained by fitting single-wavelength UV-Vis kinetics with a biexponential equation.

IsdB-Hb complex	k_1_ (s^−1^)	Amp1 (%)	k_2_ (s^−1^)	Amp2 (%)
IsdB-MetHb	0.20 ± 0.01	67	0.053 ± 0.001	33
IsdB-α-semiMetHb	0.29 ± 0.01	88	0.039 ± 0.001	12
IsdB-β-semiMetHb	0.27 ± 0.01	84	0.037 ± 0.001	16
IsdB^Y440A^-MetHb	0.22 ± 0.01	95	0.017 ± 0.001	5

### Determination of microscopic kinetic constants of complex formation

Literature data report about the affinity of the IsdB:Hb complex^12,20^, structure of IsdH and IsdB hemophores in complex with Hb, also with isolated domains^27,32^, and mechanism/kinetics of heme transfer^15,24,25,31,33–35^. However, key information is missing on the dynamics of complex formation and on the subtle mechanisms that control the kinetics of the heme transfer. SPR experiments were carried out on different IsdB (wt and Y440A mutant) and Hb variants (OxyHb, MetHb and semiHbs) under the same experimental conditions, to define the simplest kinetic model and determine the microscopic rate constants for the individual steps.

Also based on literature data on similar systems^24^, a general kinetic model for complex formation with Hb and heme extraction can be represented as follows:

$${{\rm{IsdB}}}^{{\rm{apo}}}+{\rm{MetHb}}\rightleftharpoons {{\rm{IsdB}}}^{{\rm{apo}}}\,:\,{\rm{MetHb}}\to {{\rm{IsdB}}}^{{\rm{holo}}}\,:\,{{\rm{Hb}}}^{{\rm{apo}}}\rightleftharpoons {{\rm{IsdB}}}^{{\rm{holo}}}+{{\rm{Hb}}}^{{\rm{apo}}}$$

**Figure 3:**
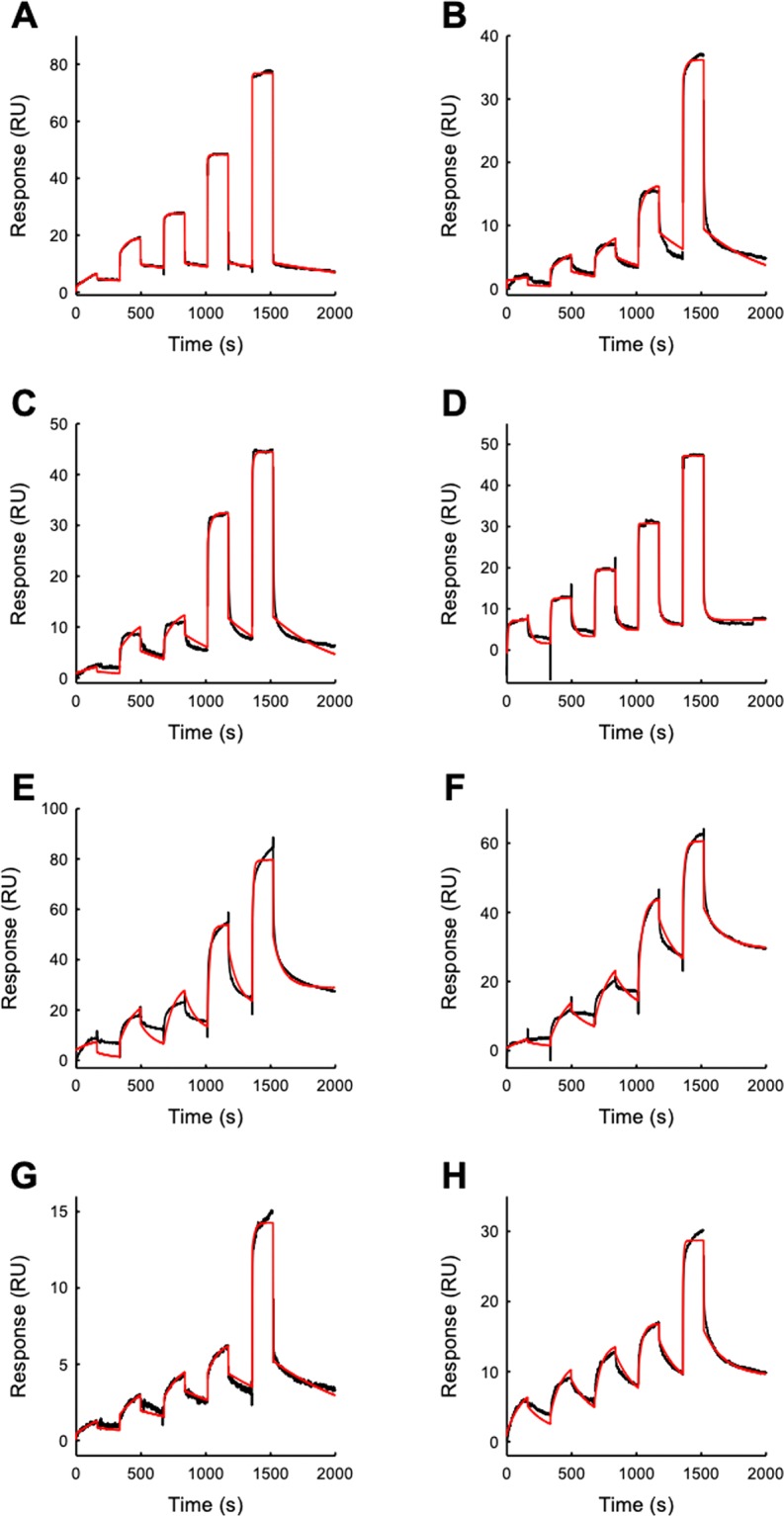
SPR sensorgrams (black curves) of the response (Resonance Units, RU) versus time of the single-cycle kinetics performed by injecting five increasing concentrations (0.01, 0.05, 0.1, 0.5, 1 µM) of Hb over the IsdB-functionalized substrate. The red curves are the fitting with either a 1:1 or a two state irreversible binding model. Fitting parameters are reported in Table 2. The resulting kinetic parameters are reported in Table 2. (**A**) IsdB-OxyHb (1:1) (**B**) IsdB-α-semiOxyHb (1:1) (**C**) IsdB-β-semiOxyHb (1:1) (**D**) IsdB-MetHb (two-state irreversible) (**E**) IsdB-α-semiMetHb (two-state irreversible) (**F**) IsdB-β-semiMetHb (two-state irreversible) (**G**) IsdB^Y440A^-OxyHb (1:1) (**H**) IsdB^Y440A^-MetHb (two-state irreversible).

IsdB forms an encounter complex with MetHb, followed by heme extraction and by complex dissociation. The heme removal step is assumed to be irreversible based on published data on IsdH^24^ and on the expected extensive conformational changes towards a partially unfolded state occurring on Hb upon heme transfer^13^. On the other hand, OxyHb binds IsdB without transferring heme (Fig. 2A, inset) and, thus, only the first, reversible step is sufficient to describe the interaction. Therefore, SPR experiments were firstly carried out on OxyHb which represents a simplified model for the IsdB:Hb interaction. The sensorgrams obtained from the successive injections of OxyHb at five progressively higher concentrations, ranging from 0.01 to 1 µM, on a IsdB-functionalized chip, are shown in Fig. 3A (black curves). The response signal increased after every injection and reached a steady state value before the end of each injection, indicating the formation of the IsdB:OxyHb complex. Subsequently, the buffer was flowed over the ligand and the response signal decreased, indicating the dissociation of Hb. A 1:1 model was used to fit the data and to extract the kinetic parameters for the IsdB:OxyHb interaction (Fig. 3A, red curve, and Table 2). Fitting to a two-state model with either a reversible or irreversible second step does not improve the fitting significantly (data not shown).

**Table 2:** Microscopic kinetic constants and dissociation constants calculated by SPR.

IsdB-Hb complex	k_on(1)_ (10^3^ M^−1^s^−1^)	k_off(1)_ (10^−3^ s^−1^)	k_on(2)_ (10^−3^ s^−1^)	K_D_ (nM)	R_max_	χ^2^
IsdB-OxyHb	8.1 ± 0.1	0.83 ± 0.02		102 ± 3	10.43	0.27
IsdB-α-semiOxyHb	44 ± 1	1.93 ± 0.05		44 ± 2	9.85	1.22
IsdB-β-semiOxyHb	77 ± 2	2.0 ± 0.1		26 ± 1	12.35	2.85
IsdB-MetHb	530 ± 40	37 ± 1	1.21 ± 0.03	70 ± 6	11.8	1.09
IsdB-α-semiMetHb	130 ± 10	14 ± 1	1.51 ± 0.03	108 ± 11	52	10.90
IsdB-β-semiMetHb	54 ± 2	4.9 ± 0.2	1.21 ± 0.03	91 ± 5	42.7	3.36
IsdB^Y440A^-OxyHb	9.7 ± 0.1	1.2 ± 0.1		124 ± 10	5.3	0.12
IsdB^Y440A^-MetHb	53 ± 1	5.1 ± 0.2	0.97 ± 0.02	96 ± 4	16.6	0.94

It has been suggested that IsdB^13,28^ could show preferences in binding to either α or β chains of Hb. This observation has critical consequences not only on the mechanistic description of complex formation, but also on the design of protein-protein interaction (PPI) inhibitors tackling the complex stability. For a direct study of any chain preferences we decided to use semiHbs, α/β heme molecule for each dimer at either the α or β subunit, hence any difference in the binding and extraction process can be directly attributed to one specific subunit. Figure 3B shows the sensorgram obtained by injecting five increasing concentrations of α-semiOxyHb over the wt IsdB-functionalized substrate. The response signal increases after every injection, witnessing the formation of the IsdB:α-semiOxyHb complex. Then, the buffer injection causes the partial dissociation of the complex. Fitting to a 1:1 model gives a good description of experimental data (chi square = 1.22; Table 2). Similar results were obtained with β-semiOxyHb (Fig. 3C, chi square = 2.85; Table 2). Interestingly, α-semiOxyHb and β-semiOxyHb show very similar microscopic rate constants and calculated K_d_, suggesting that IsdB is able to bind equally well the two globin chains. The same single-cycle kinetics SPR approach described above for the OxyHbs was used to study the interaction between IsdB and MetHb or semiHbs in the oxidized form. Figure 3D shows the sensorgram obtained by injecting five increasing concentrations of MetHb over the IsdB functionalized substrate. The sensorgrams were best fitted by using a two-state irreversible model (red line). The same fitting model was used to fit binding data with α-semiHb and β-semiHb in the MetHb form (Fig. 3E,F, respectively); the corresponding parameters are reported in Table 2. The complex formation is significantly faster for MetHb, which also dissociates faster from IsdB with respect to OxyHb. Despite the different microscopic kinetic constants, the resulting affinity of the complex is similar when either OxyHb or MetHb are used. In the model proposed, the first step, corresponding to the encounter complex formation, is followed by a second, irreversible step. As for the wild type protein, the binding kinetics of the IsdB^Y440A^ variant were analyzed using a 1:1 model for OxyHb and a two-step irreversible or reversible model for MetHb. The latter possibility was taken into account because, given the inability of the IsdB^Y440A^ mutant to complete the heme transfer, heme extraction and accompanying conformational changes could in principle be reversible. Sensorgrams are shown in Fig. 3G, H for OxyHb or MetHb binding to IsdB^Y440A^ mutant, respectively. Fitting with a 1:1 model of OxyHb binding to IsdB^Y440A^ results in kinetic parameters very similar to those obtained for wt IsdB (Table 2), demonstrating that the mutation does not affect the complex formation. Interestingly, both two-step reversible and two-step irreversible models were able to properly fit the experimental data obtained for MetHb. The two-step reversible model gives a very slow *off* rate for the second step, thus making this event virtually irreversible. As a matter of fact, the *on* rate for the second step (data not shown) is comparable to the one calculated with the two-step irreversible model that was thus chosen to fit the data.

### Molecular simulations

**Figure 4:**
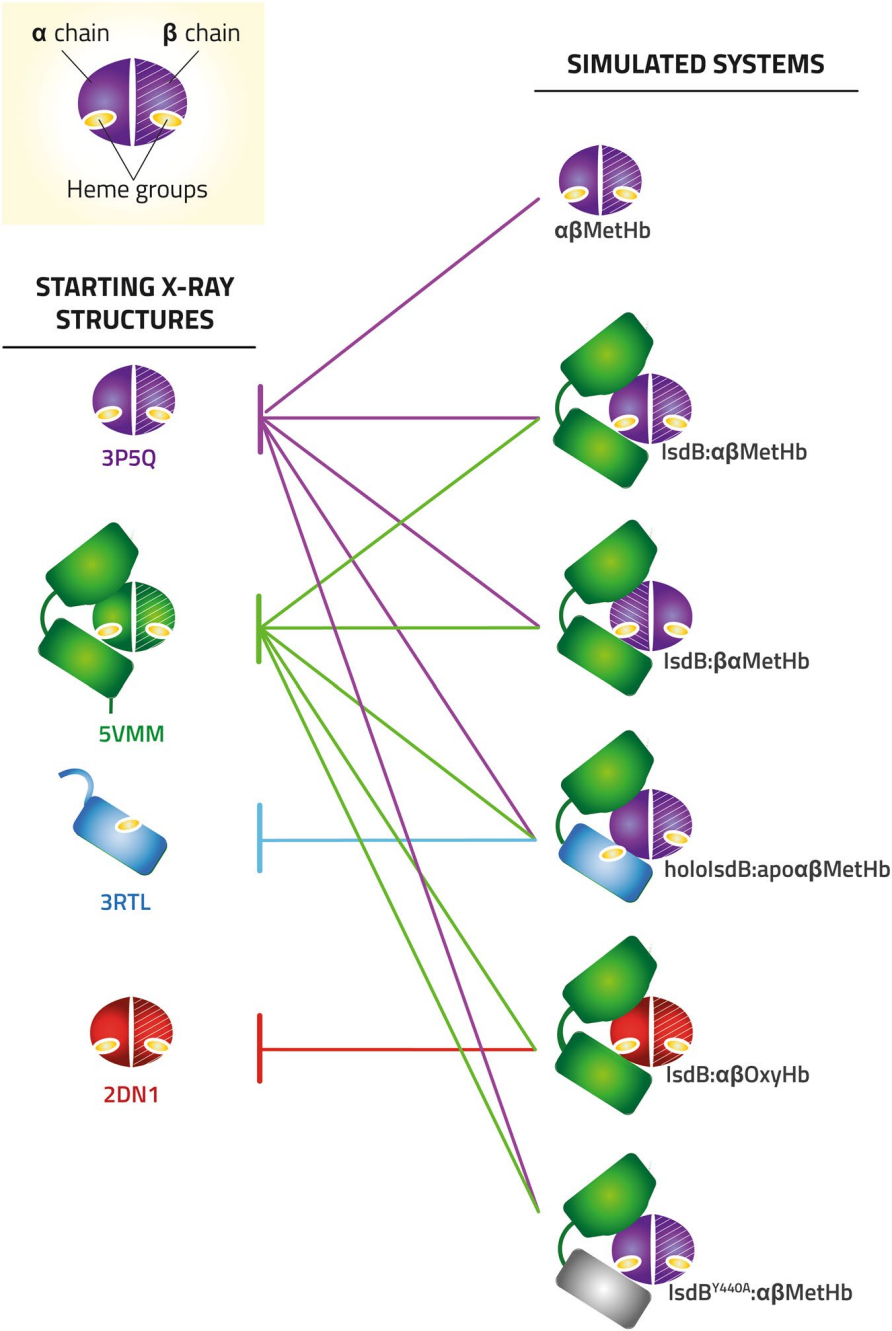
Models assembly for molecular modelling. The original X-ray structures and the corresponding PDB codes are reported on the left.

Suitable models for simulating the IsdB:Hb interaction (Figs. 1 and 4) were assembled using the unique available co-crystallized complex (PDB code 5vmm), recently reported by Bowden *et al*.^13^, where two IsdB molecules bind the α chains of a Hb tetramer, while the β chains are bound by a single N1 domain. Other structures reporting complexes of IsdH with Hb present the IsdH monomer, or the only N1, bound to one Hb dimer by means of the α chain (PDB code 4xs0 and 4fc3, respectively), or four IsdH bound to a Hb tetramer, in agreement with a 1:1 stoichiometry (PDB code 4ij2). To limit the modelling we decided to perform our simulations on the unique complete assembly, formed by one IsdB monomer and one Hb dimer.

**Figure 5:**
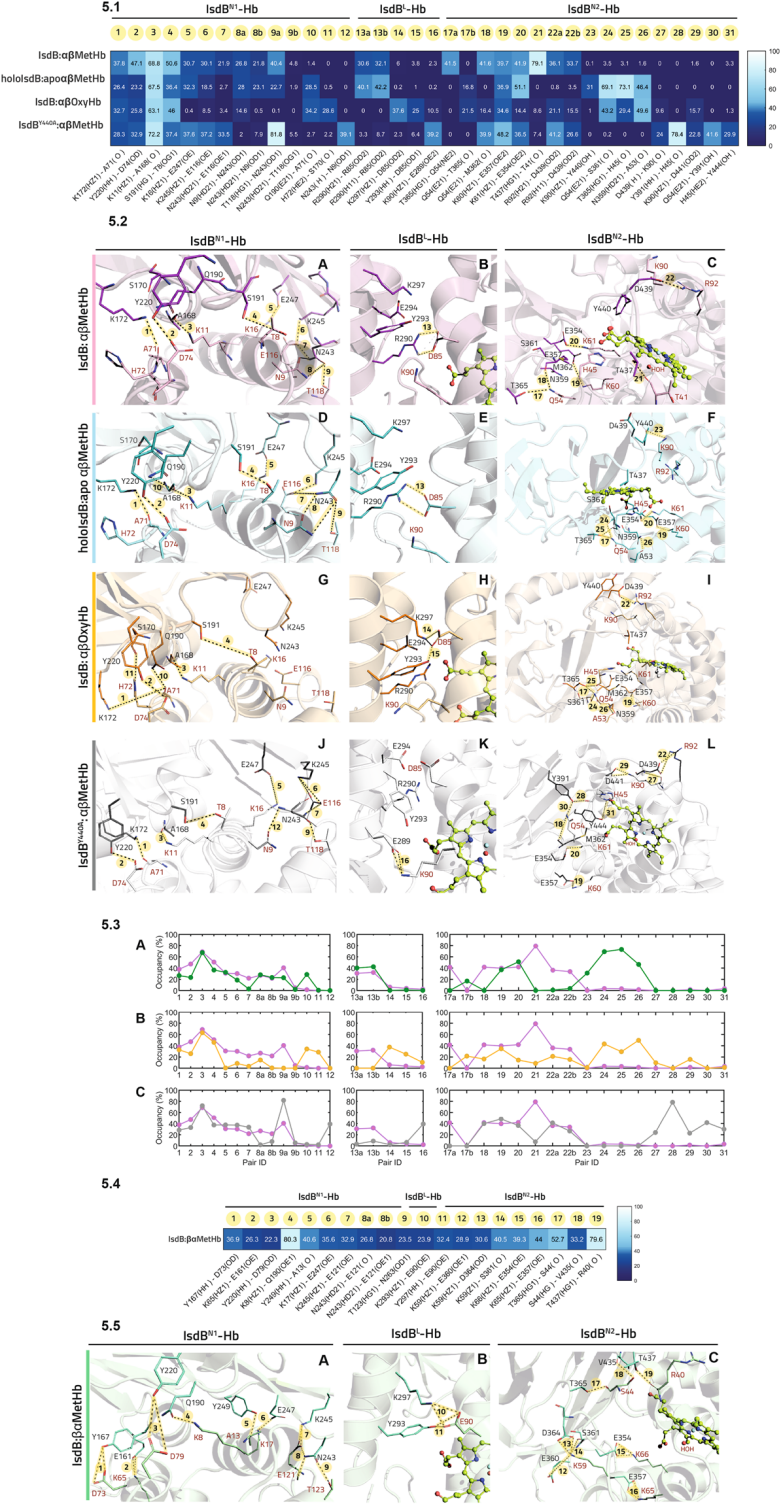
High-occupancy hydrogen bonds in IsdB:Hb. (**1**) Relevant polar contacts at IsdB:αβHb interface and relative hydrogen bond occupancy during MD simulations. Each polar contact is given an ID number. Donor-acceptor pairs having an occupancy higher than 20% are reported for each MD simulation and compared with the same pairs in all the other simulations. Polar contacts were subdivided according to their localization (IsdB^N1^-Hb, IsdB^L^-Hb, IsdB^N2^-Hb interfaces, respectively). (**2**) High occupancy polar contacts at the IsdB:αβHb interface for IsdB:αβMetHb (pink), holoIsdB:apoαβMetHb (light blue), IsdB:αβOxyHb (light orange), IsdB^Y440A^:αβMetHb (grey) simulations, respectively. Polar contacts are shown as dashed black lines, highlighted in yellow and numbered in bold, according to their ID number in Panel 1. Donor-acceptor pairs are depicted in sticks and labelled in black (IsdB residues) and in red (Hb residues). The heme group (green) is represented in spheres and sticks. (**3**) Comparison of the H-bond occupancy profile at the interface of the complexes IsdB:αβMetHb and holoIsdB:apoαβMetHb (**A**); pink and light blue line, respectively), IsdB:αβMetHb and IsdB:αβOxyHb (**B**); pink and orange line, respectively), IsdB:αβMetHb and IsdB^Y440A^:αβMetHb (**C**); pink and grey line, respectively). H-bonds are indicated by numbers according to 5.1. (**4**) Relevant polar contacts at IsdB:βαMetHb interface and relative hydrogen bond occupancy during MD simulations. Polar contacts with an occupancy higher than 20% are reported, each pair is given an ID number. (**5**) View of polar contacts at the IsdB:βαMetHb interface; each hydrogen bond is shown as a dashed line, highlighted in yellow and labelled according to the ID number in Panel 4. Hb residues are tagged in red, while IsdB residues are tagged in black.

In the 5vmm structure, the heme occupies an intermediate position between IsdB and Hb and the α chain helix F is partially unfolded. To properly simulate the initial encounter complex we replaced Hb by either MetHb or OxyHb structures (PDB code 3p5q and 2dn1, respectively; see Materials and Methods for further details). As mentioned, IsdB is formed by two NEAT domains, with N1 involved in Hb recognition, and N2 in heme extraction. The two are connected by a α-helix-based linker (IsdB^L^; Fig. 1). All elements interact with Hb α chain, at a different extent. The following systems, modelled as described in Fig. 4, were prepared and submitted to 1 μs molecular dynamics (MD) simulations (Fig. S4): MetHb, IsdB:αβMetHb, holoIsdB:apoαβMetHb, IsdB:αβOxyHb, IsdB^Y440A^:αβMetHb and IsdB:βαMetHb. Each complex was carefully investigated to identify the crucial residues involved in the complex formation and, possibly, in the heme extraction. The occupancy of the hydrogen bonds, as identified by Gromacs, formed at the IsdB^N1^-Hb, IsdB^L^-Hb and IsdB^N2^-Hb interfaces was traced along the MD trajectories and is reported in Fig. 5, along with the corresponding involved residues. Only contacts showing an average occupancy higher than 20% in one of the analyzed models have been considered relevant for the analysis.

#### IsdB:αβMetHb

The most conserved contact (number 3) at the IsdB^N1^:Hb interface is formed by Lys11^Hb^ and the carbonyl oxygen of Ala168^IsdB^. This contact shows an occupancy close to 70% in all the analyzed models, *i.e*. IsdB:αβMetHb, holoIsdB:apoαβMetHb and IsdB:αβOxyHb. Other crucial contacts in IsdB:αβMetHb model are number 1, 2, 4 and 9, all having an occupancy close or higher than 40% (Fig. 5.1). Overall, the key residues involved are Thr8^Hb^, Asn9^Hb^, Lys11^Hb^, Ala71^Hb^, Asp74^Hb^, Thr118^Hb^, Ala168^IsdB^, Lys172^IsdB^, Ser191^IsdB^, Tyr220^IsdB^ and Asn243^IsdB^ (Fig. 5.2A).

**Figure 6:**
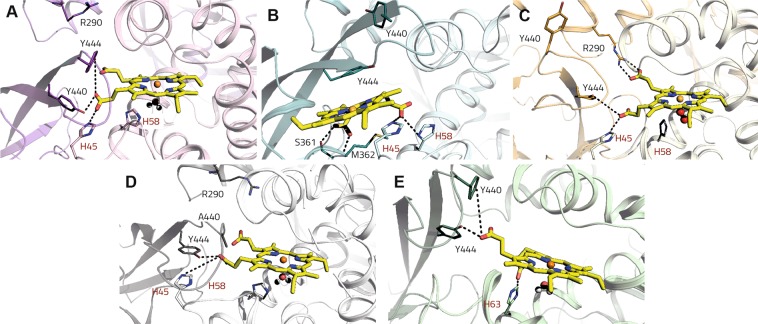
Polar contacts made by the heme with the IsdB and Hb residues for the analyzed complexes. (**A**) IsdB:αβMetHb. (**B**) HoloIsdB:apoαβMetHb. (**C**) IsdB:αβOxyHb. (**D**) IsdB^Y440A^:αβMetHb. (**E**) IsdB:βαMetHb. Hydrogen bonds are shown as dashed lines, Hb residues are labeled in red, while IsdB residues are labeled in black.

At the IsdB^L^:Hb interface we observed the formation of a salt bridge between Asp85^Hb^ and Arg290^IsdB^ (Fig. 5.2B), while at IsdB^N2^:Hb interface several H-bonds are maintained along the simulation, involving Thr41^Hb^, Lys60^Hb^, Lys61^Hb^, Arg92^Hb^, Glu357^IsdB^, Thr437^IsdB^ and Asp439^IsdB^ (Fig. 5.2C). This complex interaction pattern might properly prepare the system for the transfer of the heme, which interacts with His45^Hb^, His58^Hb^, Tyr440^IsdB^ and Tyr444^IsdB^ (Table S1, Fig. 6A). These last two residues are likely involved in pulling the heme out of its binding pocket. However, in this and in the following simulations, no significant translation of the heme can be observed.

#### holoIsdB:apoαβMetHb

We performed the same analyses on the holoIsdB:apoαβMetHb model, which should represent the final step of the IsdB:Hb interaction, upon the heme transfer. We have to consider that, if Hb is released by IsdB once the heme transfer has occurred, this state could represent a poorly populated species (see discussion). Even so, we decided to model this state to have indications about the rearrangement of the complex interface.

The IsdB^N1^:Hb (Fig. 5.2D) and IsdB^L^:Hb (Fig. 5.2E) interfaces are quite similar to those described above for IsdB:αβMetHb, while the IsdB^N2^:Hb line significantly differs (Fig. 5.2F). In particular, contacts involving Gln54^Hb^ and Ser361^IsdB^, His45^Hb^ backbone and Thr365^IsdB^, Ala53^Hb^ and Asn359^IsdB^ (contacts number 24, 25 and 26, respectively) are formed and maintained.

With respect to IsdB:αβMetHb, the propionates of the heme, now located in the IsdB binding site, still interact with His45^Hb^ and His58^Hb^ on Hb, and with Ser361^IsdB^ and Met362^IsdB^ (as already reported in^22^) on IsdB (Table S1, Fig. 6B).

#### IsdB:αβOxyHb

The H-bond pattern at the IsdB:αβOxyHb interface is still quite different from IsdB:αβMetHb. We have to remember that the dissociation constant for the two complexes is similar (100 ± 10 nM and 69 ± 1 nM, respectively. See Table 2), but the heme cannot be extracted from OxyHb. At the IsdB^N1^:Hb level six contacts maintain an occupancy higher than 20%, with the most relevant being formed by Asp74^Hb^ and Tyr220^IsdB^, Lys11^Hb^ and Ala168^IsdB^, Thr8^Hb^ and Ser191^IsdB^ (Fig. 5.2G). At the IsdB^L^:Hb interface the Asp85^Hb^-Arg290^IsdB^ contact observed in IsdB:αβMetHb and conserved in holoIsdB:apoαβMetHb, is lost and replaced by Asp85^Hb^-Tyr293^IsdB^ and Asp85^Hb^-Lys297^IsdB^ interactions (Fig. 5.2H). Finally, at the IsdB^N2^:Hb line, the highest occupancy contacts are Gln54^Hb^-Ser361^IsdB^ and Ala53^Hb^-Asn359^IsdB^(number 24 and 26, respectively. Fig. 5.2I). Interestingly, the heme strongly interacts with Arg290^IsdB^, previously involved in stabilizing the IsdB^L^:Hb interface, while the contacts with His45^Hb^, Tyr440^IsdB^ and Tyr444^IsdB^, even if still present, are less conserved, possibly disrupting the proper residue arrangement for heme extraction (Table S1 and Fig. 6C).

#### IsdB^Y440A^:αβMetHb

The IsdB^N1^:Hb interface of the Y440A mutant presents a similar profile to that of IsdB:αβMetHb (Fig. 5.1, 5.2L). A comparable number of interactions is maintained, as well as the crucial residues involved. The IsdB^L^:Hb interface presents a single contact involving Lys90^Hb^ and Glu289^IsdB^, thus being less stable than in the previous cases (Fig. 5.2M), in which a salt bridge or multiple contacts were formed, as also confirmed by the following ED analysis. At the IsdB^N2^:Hb line, while a number of interactions are maintained as in the wild type complex, additional contacts appear, e.g. His45^Hb^-Tyr444^IsdB^ and Gln54^Hb^-Tyr391^IsdB^ (Fig. 5.2N). The heme also maintains a weak interaction with His45^Hb^ and a more stable one with Tyr444^IsdB^, while the two residues are not involved in interacting one with each other (Table S1, Fig. 6D).

In Fig. 5.3 we have compared the H-bond occupancy profile of the IsdB:αβMetHb complex with that of holoIsdB:apoαβMetHb (Fig. 5.3A), IsdB:αβOxyHb (Fig. 5.3B) and IsdB^Y440A^:αβMetHb (Fig. 5.3C). Interestingly, with a few exceptions, the trend at the IsdB^N1^-Hb level is rather maintained. This confirms the role played by the aforementioned residues and suggests that, regardless the ability to complete heme transfer, the key interactions are maintained.

The contacts at the IsdB^L^-Hb line are much more variable. While the profile is maintained in both IsdB:αβMetHb and holoIsdB:apoαβMetHb (Fig. 5.3A), in the IsdB:αβOxyHb and IsdB^Y440A^:αβMetHb cases different residues play key roles in contact formation (Fig. 5.3B,C respectively).

At the IsdB^N2^-Hb interface the pattern presented by IsdB:αβMetHb cannot be reproduced by any of the other complexes. The only conserved contact (number 19) involves Glu354^IsdB^ and Lys61^Hb^, also maintained in the IsdB:βαMetHb model (number 13, Fig. 5.4). The profile of holoIsdB:apoαβMetHb is, instead, similar to that shown by IsdB:αβOxyHb. In both cases, the heme cannot be extracted and contacts between Met362^IsdB^ and Thr365^IsdB^ with Gln54^Hb^, or Thr437^IsdB^:Thr41^Hb^ and Asp439^IsdB^:Arg92^Hb^ are lost. The IsdB^Y440A^:αβMetHb profile is slightly more similar to that of IsdB:αβMetHb, at least in the first part, while additional interactions are formed by the residues at the IsdB carboxylic terminal. We should remember that the mutated IsdB is not able to complete the heme extraction^22^. The only contact maintained in all the simulations involves His45^Hb^, while heme extraction is mainly assisted by Tyr440^IsdB^ and Tyr444^IsdB^, highlighting their role in pulling the heme out of its binding site (Fig. 6).

#### IsdB:βαMetHb

Kinetic data indicate that the dissociation constants for the first reversible binding step of IsdB to Hb are similar when calculated with α-semiHb and β-semiHb, suggesting a similar binding of IsdB to both α and β chains (Table 2). To further investigate α/β selectivity, we modelled an IsdB:Hb complex in which Hb chains are inverted and the IsdB directly binds to the β chain (IsdB:βαMetHb, Fig. 4). The H-bond occupancy analysis showed that similar contacts are maintained with respect to the IsdB:αβMetHb complex (Fig. 5.4). The most relevant at the IsdB^N1^:Hb interface are Asp79^Hb^:Tyr220^IsdB^, Glu121^Hb^:Lys245^IsdB^, Glu121^Hb^:Asn243^IsdB^, Lys17^Hb^:Glu247^IsdB^ (also conserved in IsdB:αβMetHb), Lys8^Hb^:Gln190^IsdB^ and Asp73^Hb^:Tyr167^IsdB^ (Fig. 5.5A). The IsdB^L^:Hb interface is stabilized by additional interactions in which Glu90^Hb^, corresponding to Asp85^Hb^ in the α chain, contacts Tyr293^IsdB^ and Lys297^IsdB^ (Fig. 5.5B). Finally, at the IsdB^N2^:Hb line we can observe the Lys65^Hb^:Glu357^IsdB^ and Lys66^Hb^:Glu354^IsdB^ contacts, also present at the IsdB:αβMetHb interface, the additional Arg40^Hb^:Thr437^IsdB^ and those involving Lys59^Hb^, interacting with Glu360^IsdB^, Ser361^IsdB^ and Asp364^IsdbB^ (Fig. 5.5C). The heme propionates are weakly coordinated by His63^Hb^ and Tyr440^IsdB^ and more constantly by Tyr444^IsdB^ (Table S1, Fig. 6E). These findings further support the capability of IsdB to similarly interact with both Hb chains and to, consequently, extract the heme.

**Figure 7:**
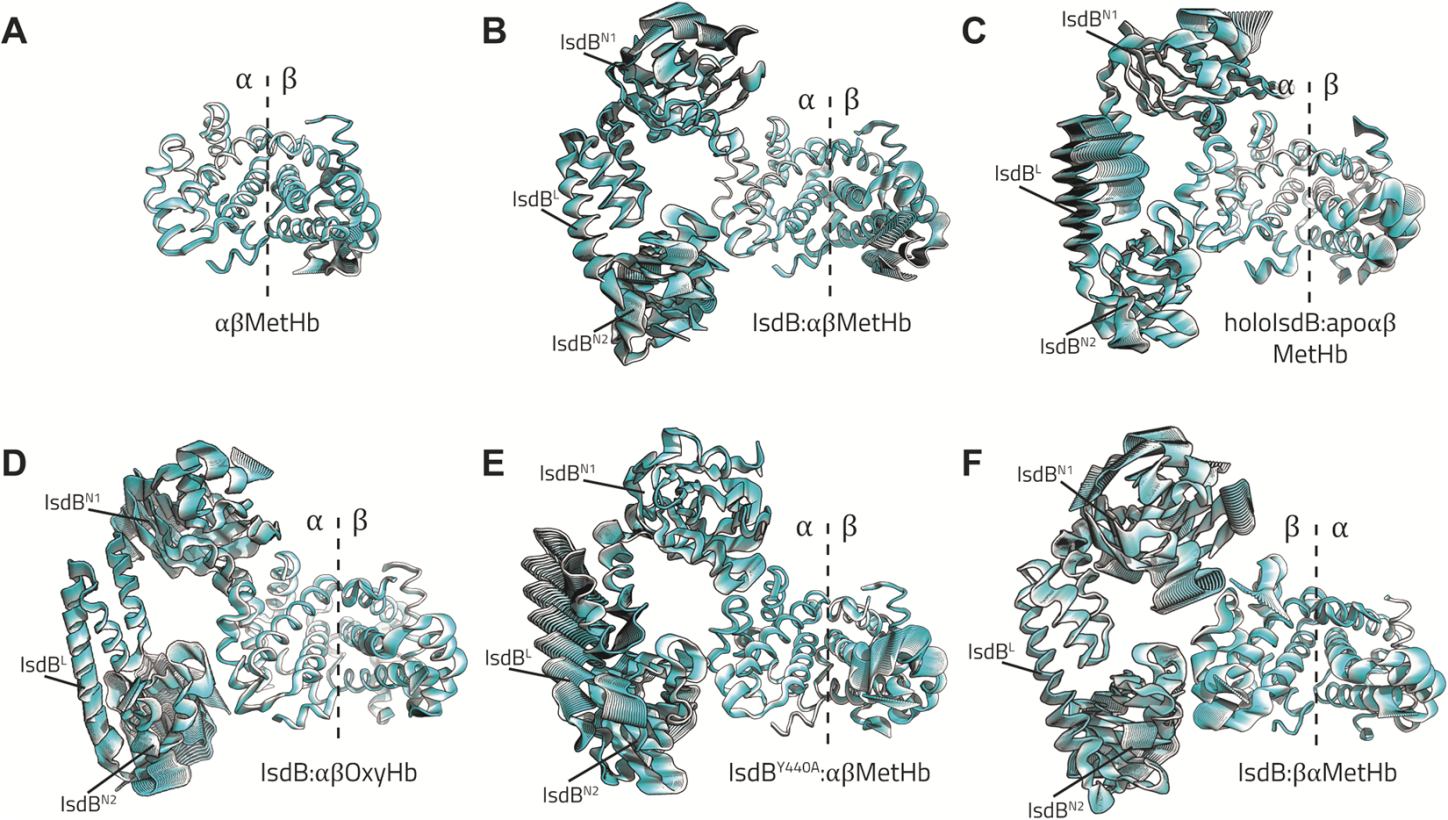
Extreme trajectory conformations projected along the first eigenvector.

To get insights about the complex intrinsic flexibility we analyzed all MD trajectories by means of Essential Dynamics (ED), able to identify the essential motions of the protein backbone. The well-known flexible C/E and C/D loops in Hb α chain and β chain^36^, respectively, were removed from the ED analyses to not bias the identification of other dynamic elements. In all complexes, the ED was carried out separately for each individual α and β globin chain, for the αβ dimer (Hb) and for IsdB. Eigenvectors were then sorted out in decreasing order depending on their corresponding eigenvalues, which have been reported in Table S2. In Fig. 7 we graphically represented the projection of the trajectory on the first eigenvalue, responsible for the highest structural variation.

We chose as reference MetHb, whose ED shows a quite stable structure, with the highest flexibility localized at the terminal region of helix F and at the E/F loop of chain β, being notably more flexible than α chain^36^ (Fig. 7A). Hb dynamics increases upon binding of IsdB, in particular at the β chain level, in agreement with its higher flexibility and with the constraint exerted by IsdB on α chain (IsdB:αβMetHb complex, Fig. 7B). IsdB shows, as well, a significant level of flexibility in the two NEAT domains and a little less in the linker section. When the heme has been already transferred to IsdB, the entire complex flexibility decreases, with the only exception of the IsdB linker (holoIsdB:apoαβMetHb*,* Fig. 7C). Indeed, the first eigenvector for IsdB mostly regulates the flexibility of the tether between N1 and N2. In the IsdB:αβOxyHb complex the IsdB dynamics increases (first eigenvalue equal to 12,4 nm^2^, Table S2), in particular at N2 domain level (Fig. 7D). Analogously, a high plasticity of N2 can be observed for IsdB^Y440A^:αβMetHb (first eigenvalue for IsdB equal to 18.3 nm^2^), in which the heme extraction is not complete. As well, the linker shows the highest flexibility, according to the projection on the first eigenvector (Fig. 7E, Table S2). Finally, in the IsdB:βαMetHb (Fig. 7F) complex the IsdB linker and N2 domain show a flexibility level similar to that observed for IsdB:αβMetHb, while the N1 domain seems quite disordered. The plots reporting the trajectory projection on the first two eigenvectors, defined as the essential space, are reported in Fig. S5.

To further investigate the system dynamics, we calculated the cosine content for the first eigenvector (*c*_*i*_; Table S3), giving indications about the possible functional main movements far from random diffusion. Overall, the hemoglobin dimer (Hb) shows a high *c*_*i*_ (*e.g*. non-functional movements) in IsdB^Y440A^:αβMetHb and in IsdB:αβOxyHb complexes, where IsdB is defective or is unable to conclude the heme extraction. Similarly, the *c*_*i*_ is high for the Hb in MetHb, consistently with the absence of the hemophore.

Even more interestingly, the ED shed light on structural rearrangements (*c*_*i*_ ≤ 0.1) on IsdB taking place in complexes where the heme transfer is hindered. Specifically, in holoIsdB:apoαβMetHb and IsdB^Y440A^:αβMetHb, where the heme transfer already occurred or will not be completed, the first eigenvector indicates a massive motion involving the linker domain, pulling it away from hemoglobin. Correspondingly, in IsdB:αβOxyHb, where the extraction cannot be performed, the linker slides down towards the IsdB^N2^ domain. On the other hand, no such motions have been registered in IsdB:αβMetHb and in IsdB:βαMetHb, for which the high cosine content reflects the absence of large non-equilibrium transitions (Fig. S6).

## Discussion

Heme acquisition by pathogens using specialized receptors and membrane transporters has attracted much attention since this constitutes a relevant process for bacterial physiology and virulence and, therefore, a potential promising site of therapeutic intervention. Although many reports demonstrate the larger relevance of IsdB in supporting *S. aureus* iron demand during infection, the IsdB system is far less characterized than IsdH, limiting the understanding of the reasons for redundancy of these Hb receptors and, eventually, the possibility to envisage approaches for interfering with complex formation.

IsdB purified from *E. coli* recombinant systems contains a variable fraction of heme bound, ranging from 1 to 30%, depending on the strain used for the expression, the expression yields and the growth conditions^37^. The fraction of holoIsdB present in the IsdB preparations used to study the interaction with Hb and heme extraction is rarely mentioned and quantified. The knowledge of the fraction of heme in the starting preparation is relevant, since it might affect Hb binding and introduce artifacts in the calculation of affinity and/or heme transfer efficiency. The amount of holoIsdB in all the preparations used in this study was calculated on the basis of the extinction coefficient of the Soret band and is consistently lower than 5%. The low amount of heme-saturated IsdB obtained from bacterial cultures grown in rich medium depends on the high expression yields of this expression system and the limiting heme availability in *E. coli*. The negligible amount of holoIsdB in the preparation and hence the knowledge of the exact apo-protein concentration allowed to accurately calculate the stoichiometry of Hb binding to IsdB in solution. Such stoichiometric ratio is 1, *i.e*. each Hb monomer binds one IsdB molecule. A further point that needed to be addressed before starting any mechanistic and kinetic investigation of complex formation and heme extraction process is the oligomeric state of Hb under our experimental conditions. It is well assessed that human Hb is a stable tetramer only in concentrated solutions, like those found inside the erythrocytes, and that once the concentration drops to the low micromolar range a mixture of dimers and tetramers is present. Dimers are stable down to low nM concentrations, when monomerization occurs. Relevant for the current study, the quaternary state of Hb affects the rate of spontaneous heme release^38^. We calculated a K_D_ for the tetramer/dimer equilibrium of 0.25 ± 0.05 µM for OxyHb and 0.52 ± 0.06 µM for MetHb (Fig. S2). Therefore, under the conditions used for the stoichiometric titration, Hb is expected to be 90% in the tetrameric form, which might cause steric hindrance in binding of four IsdB molecules^13^. However, it is conceivable that, as already reported for IsdH^N2N3^^28^, IsdB binding promotes Hb dimerization and that the dominant form of the complex is that where two IsdB chains are bound for each Hb dimer. As mentioned, the 1:1 stoichiometry agrees with the published IsdB:MetHb complex, where the Hb tetramer is bound to two full-length IsdB chains and two isolated N1 domains, likely generated by proteolysis during crystallization^13^. A similar stoichiometry (3.5 hemophore chains per Hb tetramer) was observed for the IsdH:MetHb system published by Dickson *et al*. in 2014 (PDB code 4ij2)^27^.

In the concentration range used for SPR experiments Hb is mainly dimeric, from 98% at 0.01 μM to 35% at 1 μM. These experimental conditions also span the physiological range usually observed for extracellular Hb, falling in the low micromolar range. The models used to fit SPR data were chosen on parsimony basis and are consistent with previous reports on similar systems^39^. The two-step model used for MetHb allows to separate the contributions to the overall process of the bimolecular binding step and the following event(s). The bimolecular rate of complex formation with IsdB is consistently higher for MetHb than OxyHb. However, the encounter complex also dissociates faster, and thus the resulting affinity is similar for OxyHb and MetHb. The kinetic control of protein-protein association is a well-assessed strategy to achieve interaction specificity, for example in the case of different proteins competing for the same receptor^40^. In the case of IsdB the faster association rate with MetHb compared to OxyHb might be suggestive of some kinetic bias for complex formation with the productive species.

Of notice, the K_D_ for bimolecular complex formation is similar when binding occurs at the α or β subunits, *i.e*. when α- and β-semiHb are used. This unambiguously supports the view that IsdB does not show any chain preference in binding to Hb. The equivalence of α and β subunits for IsdB binding has already been described in previous reports that, however, exploited less direct approaches, *i.e*. the observation of mono-exponential heme-transfer kinetics or the analogy with IsdH^13,24–26,28,41^. SPR has already been applied for determining the affinity of *S. aureus* hemophores to Hb; however, data analysis was limited to the thermodynamics of complex formation, with no kinetic insight. The dissociation constants reported for IsdH^12,33,42^ and IsdB^12,37^ fall in the nanomolar range independently from the chip architecture and the immobilization tags and are thus comparable to those found in this report^12,33^. With MetHb, the encounter complex formation is followed by a slow, irreversible step. This step might be attributed to heme transfer and/or an associated, rate-limiting conformational change of the protein. However, if the k_on_ for the second step is compared with the k_obs_ obtained by UV-Vis absorption spectroscopy (Fig. 2) or with published data^13,20,24,31,34^, it is 2-order of magnitude slower. Indeed, the calculated rate is comparable to or slightly faster than the rate of spontaneous release of heme from Hb depending on the subunit and the oligomeric state considered^38^. This is apparently in contrast with other reports, where a “catalyst” role was attributed to IsdB and other proteins involved in heme transfer from Hb to the bacterial cell^20,31^. That assumption was based on the observation that, in the presence of IsdB, heme is transferred from Hb to IsdA 100-fold faster with respect to spontaneous heme release^20^. However, it should be noted that our experiments were carried out in the absence of an acceptor molecule (*i.e*.: IsdA). Our results suggest that the rate limiting step in the absence of a heme acceptor like IsdA is not heme transfer (rate-limited by a still poorly defined Hb structural isomerization), but IsdB:Hb complex dissociation:

A slow phase also characterizes the association of Hb with IsdB^Y440A^, a mutant missing a key residue for heme coordination. Consistently with the observed time-dependence of spectroscopic variations (Fig. 2C, inset), this mutant is able to initiate heme extraction from Hb, likely the trigger for complex dissociation, but appears unable to complete heme transfer.

Extensive molecular simulations were carried out to gain structural resolution both on PPI and the following dynamics. The composite pattern of electrostatic interactions that we observed might provide the IsdB:αβMetHb complex with the sufficient stability at the IsdB^N1^:Hb interface to allow the N2 domain approaching the heme binding site and starting the extraction process. Specific key residues on Hb and on the three IsdB domains (N1, N2 and the linker region) can be identified and retained relevant for the process. At the IsdB N1 level, Ala168^IsdB^, Lys172^IsdB^, Ser191^IsdB^, Tyr220^IsdB^, Asn243^IsdB^ seem implicated in the stabilization of the, likely, first interaction between IsdB and Hb. As well, crucial residues on Hb are Thr8^Hb^, Asn9^Hb^, Lys11^Hb^, Ala71^Hb^, Asp74^Hb^ and Thr118^Hb^. The role of Thr8^Hb^, Lys11^Hb^ and Asp74^Hb^ has been already highlighted in previous works by Dickson *et al*.^27^ and Pishchany *et al*.^15^ Also, Choby and co-workers recently reported that the Thr8Lys mutation reduces the binding by *S. aureus* and that the N-terminal helix of Hb α chain gives a significant contribution to the human-specific Hb recognition by the bacterium^43^. In our simulation Asn9^Hb^ is involved in contacting Gln243^IsdB^ in all but the IsdB:αβOxyHb complex. The Asn9His mutation has been also found responsible for a decreased binding. On the IsdB side, the Gln162-Ser170 region (loop 2) in N1 domain has been proved critical for Hb binding^15,20,25,41,43^ and it contains the Hb-binding motif. We specifically recognized the importance of Ala168^IsdB^ in contacting Lys11^Hb^ at the level of IsdB^N1^-αHb interface, and of Tyr167^IsdB^, interacting with Asp73^Hb^ at the IsdB^N1^-βHb interface. Other residues retained particularly relevant for the complex formations, as Phe164^IsdB^ and Tyr165^IsdB^, could be involved in forming hydrophobic interactions with Hb and, mostly, in stabilizing the helix conformation of the loop, fundamental for Hb recognition and binding^25^. While disordered in solution, the loop 2 assumes, in fact, a stable helix conformation in X-ray structures, according to a folding-upon-binding model, often observed for intrinsically disordered protein regions^25,44^. The IsdB linker is also involved in significant interactions with Hb, as previously mentioned, and also highlighted by Fonner *et al*., who found that the linker domain increases the affinity of IsdB^N1^ for MetHb and that it accelerates the heme transfer when combined with IsdB^N2^^25^. Our simulations identified Arg290^IsdB^-Asp85^Hb^ as crucial contact for the stabilization of the IsdB:αβMetHb complex and Tyr293^IsdB^-Glu90^Hb^ and Lys297^IsdB^-Glu90^Hb^ for the IsdB:βαMetHb one. The most relevant residues involved in the heme extraction at the IsdB^N2^:αHb interface appear to be, definitely, Tyr440^IsdB^ and Tyr444^IsdB^, both involved in pulling the heme out of the Hb binding site and in stabilizing it in the IsdB pocket. The inability of the IsdB^Y440A^ mutant to complete the heme extraction, as well as crystallographic evidences^22^ clearly supports the data previously reported in literature^13,22^. Our simulations found in Tyr444^IsdB^ a key residue for heme binding, with an occupancy of the H-bonds between the residue side chain and the heme propionates of about 40% (Table S1). Interestingly, His45^Hb^ has been also found relevant in contacting the heme in all the simulations, suggesting to have a relevant role in holding the cofactor and accompanying its transfer from Hb to IsdB. Once in IsdB binding site, the heme is then stabilized by Ser361^IsdB^, as also found in the transient IsdB:Hb complex^13^ and in the sole IsdB NEAT2 structure^22^. The stabilization of the IsdB^N2^:αHb is mainly achieved by Arg31^Hb^, Ser44^Hb^, Lys59^Hb^, Lys66^Hb^ on Hb α chain and, on IsdB, by Glu354^IsdB^, Glu357^IsdB^, Met362^IsdB^, Thr365^IsdB^, Thr437^IsdB^, Asp439^IsdB^, often involved in contacting also the β chain, according to our simulations (Fig. 5). None of the IsdB mentioned residues has been found mutated in a set of 3277 *S. aureus* strains taken from the NCBI SRA database, thus confirming their relevance for a stable Hb binding^15^, and the possibility to be targeted in the search for PPIs inhibitors.

With respect to the MetHb by itself, all the inspected complexes showed a more flexible Hb. The only exception is represented by the IsdB:αβOxyHb complex, in agreement with the faster microscopic kinetic constants calculated through SPR experiments for MetHb with respect to OxyHb. Interestingly, the dynamics of chain β significantly exceeds that of chain α in all the cases. Previous laser flash photolysis experiments reported the capability of the β subunit, with respect to the α one, to faster release small ligands^45,46^, with a possible implication of a different intrinsic dynamics. Furthermore, the diverse flexibility of α and β chains is also reflected in very different rates of spontaneous heme release from Hb, with β of spontaneous heme release from Hb, with β chains releasing it about 50-fold fastere than α chains. The higher degree of β flexibility is already visible in MetHb alone (Fig. 7A). The retention of this asymmetry upon IsdB binding suggests that the whole process of binding and heme extraction is unaffected by intrinsic difference in globin chain dynamics. This is in line with the observation that IsdB has a catalytic effect on heme release from Hb and that this effect overwhelms any heterogeneity in the rate of spontaneous release.

**Figure 8:**
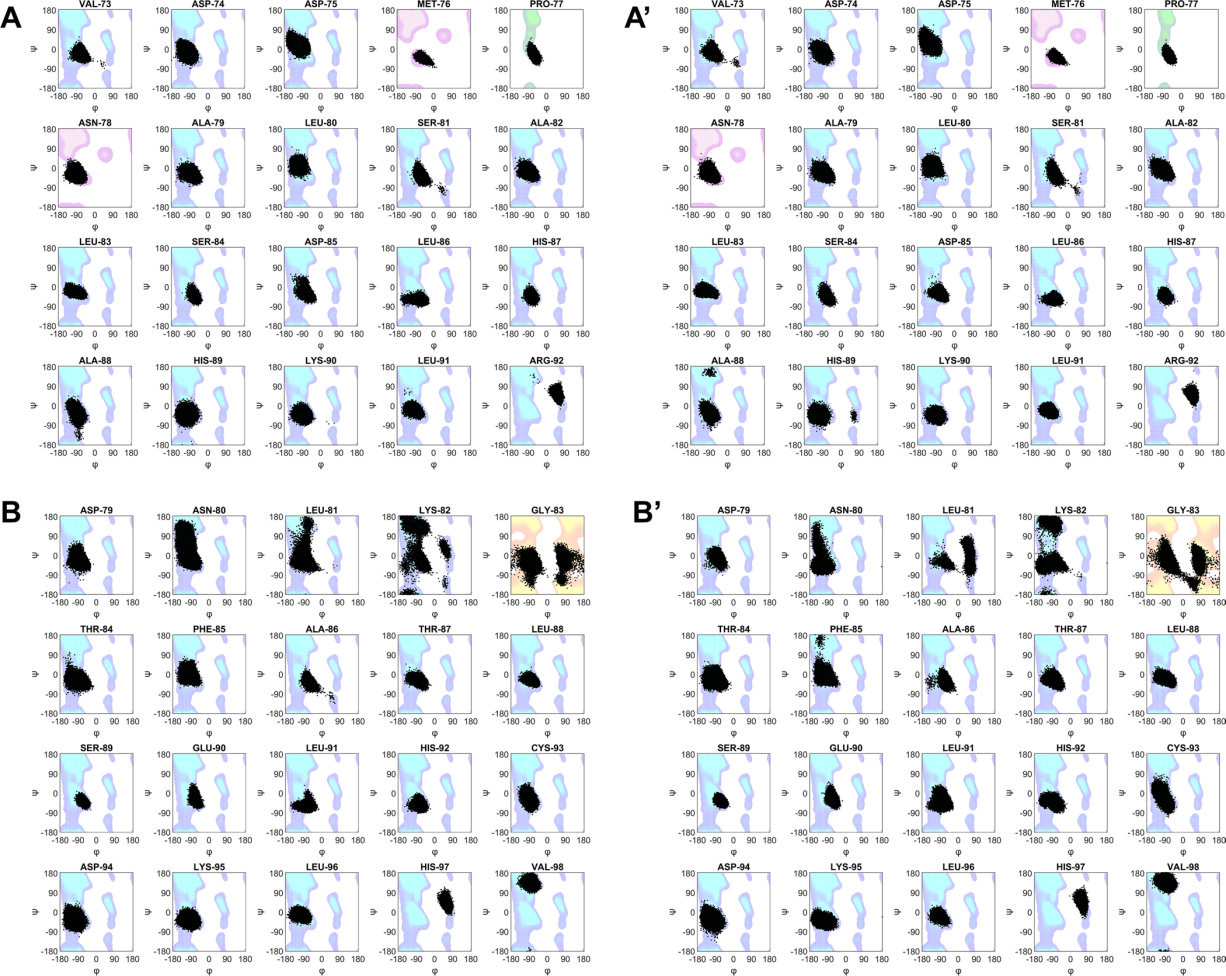
Torsion angles in F helix. Phi (φ) and psi (ψ) torsion angles of helix F were measured for each frame of the trajectories. Each measurement is represented as a black dot with coordinates (φ, ψ), and scatter plotted against the Ramachandran plot (blue contour for general, pink for pre-proline, green for proline, yellow for glycine residues, respectively). (**A**) Torsion angles of α chain F helix in αβMetHb. (**A’**) Torsion angles of α chain F helix in IsdB:αβMetHb. (**B**) Torsion angles of β chain F helix in αβMetHb. (**B’**) Torsion angles of β chain F helix in IsdB:βαMetHb. Ramachandran plots were generated in MATLAB 2019a from a dataset of high-resolution X-ray structures by Lovell *et al*.^71^, torsion angles were calculated with the g_rama_mpi function implemented in GROMACS ver. 4.6.1.

The step of heme extraction (about 0.3 s^−1^)^13,20^ seems to be represented by a large, irreversible conformational change not yet thoroughly characterized. We might argue that the increased flexibility of Hb observed along the MD simulations could correspond to the first step of this conformational adjustment, facilitating the heme scavenging. The latter could be also promoted by the complete or partial unfolding of helix F, and by the consequent weakening of the coordination bond between the heme iron and the proximal histidine. Even if no breakage of the coordination bond can be observed with such plain MD simulations, the helix undergoes a partial destabilization in both IsdB:αβMetHb and IsdB:βαMetHb models, with respect to single MetHb. This can be clearly appreciated by the decreased occupancy of intra-chain H-bonds reported in Fig. S7, and by the change in the φ/ψ torsional angle distribution observed upon the binding of the hemophore shown in Fig. 8. In both cases, the Ramachandran plot of the residues forming the helix present a change of the dihedrals distribution for the proximal histidine (His87 in α chain and His92 in β chain), the residues close the histidine and, in particular for the β chain, for the amino-terminal of the helix. This structural destabilization, induced by IsdB that pulls Hb helix F by means of Arg290^IsdB^, located in the linker region, has already been pointed out^13^. Here, a further level of detail is added and our results point to larger conformational changes involving both proteins. The ED analyses also highlighted the overall flexibility of IsdB and the importance of the linker in regulating the communication between the two NEAT domains (Fig. S6, Table S3). As suggested by the cosine content calculation, IsdB undergoes anharmonic motions, specifically located at the linker level, when the heme extraction process cannot be completed or has already happened and the entire IsdB has, reasonably, to detach from the Hb.

## Conclusions

We investigated by spectroscopic and analytical methods several aspects of IsdB:Hb complex formation that remained elusive so far, and that could be key to the design and selection of specific PPI inhibitors with a potential as innovative antimicrobials. Molecular modelling and essential dynamics were exploited to gain structural resolution, often missing or controversial, on PPI interfaces and the dynamics triggered by complex formation and leading, with MetHb but not with OxyHb, to heme extraction and transfer to IsdB. In particular, by exploiting the semiHb variants, we unequivocally demonstrated that the hemophore does not exert any selectivity in binding and extracting heme from the α or β subunit of human Hb. This is also highlighted by MD simulations and essential dynamics analysis, which showed the persistence of similar contacts at the IsdB:αβMetHb and IsdB:βαMetHb interface, and a similar level of flexibility at the IsdB linker and N2 domain. Consistently, any Hb intradimer communication following binding to the hemophore, as detected by essential dynamics, does not elicits cooperativity in Hb:hemophore binding, while could perhaps be relevant in promoting Hb monomerization. Also, microscopic kinetic rates were determined for IsdB binding to the α and β subunits of Hb, either in the oxidized form, the dominant one in the plasma under infection conditions *in vivo*, or in the reduced, oxygenated state. Spectroscopic, SPR and computational results are consistent with a model where formation of an IsdB:MetHb encounter complex is followed by heme extraction, structural strain in Hb liganded subunits, heme transfer to IsdB and a slow release of apoHb from holoIsdB. In the absence of a final acceptor of heme, such as IsdA, the whole process is limited by the slow release of apoHb from the complex with IsdB.

## Materials and Methods

### Reagents and solutions

Chemicals were purchased from Merck and Applichem if not otherwise stated. Reagents were used as received. The experiments were carried out in reaction buffer, Tris/HCl 100 mM, NaCl 150 mM, EDTA 1 mM, pH 8.0.

### Protein expression and purification

#### IsdB and IsdB^Y440A^

The gene from *S. aureus* corresponding to IsdB NEAT1 and NEAT2 domains separated by an α–helical linker (UniProt entry Q8NX66, residues 125–485) was cloned in the pASK-IBA3plus^®^ vector (IBA Lifesciences) for its expression in *E. coli* as a fusion protein with a C-terminal Strep-tag^®^II. The protein was purified by affinity chromatography on a StrepTactin^®^ resin (IBA Lifesciences). The protein was expressed and purified in high amounts, with a final yield of more than 100 mg/L of cell culture. The site-directed mutant Y440A (IsdB^Y440A^) was prepared with standard mutagenesis techniques^47^ and purified to homogeneity by the method optimized for the wild type protein.

#### Hemoglobin and semi-hemoglobins

Human hemoglobin A (Hb) was purified from outdated blood obtained from a blood transfusion center. In particular, only hemoglobin from non-smoking donors was purified, as described in^48^. The purified protein in the oxy form was aliquoted, flash-frozen in liquid nitrogen and stored at −80 °C until further use. MetHb was obtained by heme oxidation in the presence of potassium ferricyanide. Human α and β semihemoglobins (α-semiHb and β-semiHb) were prepared as described in^29^ by mixing isolated α and β chains with apoHb^49^. Briefly, heme groups were removed by 1:1 liquid-liquid extraction in butanone of an aqueous solution of 1 mM Hb, pH 2.5 with 1.0 M HCl. The extraction was repeated three times on the recovered aqueous phase. Before each extraction, the solution was centrifuged to discard the precipitated protein, and the pH adjusted to 2.5 with 1.0 M HCl. The final Hb solution was first dialyzed in distilled water to remove the excess of butanone dissolved in the aqueous phase, and subsequently in 20 mM potassium phosphate, pH 7.0, and 100 mM potassium phosphate, 150 mM NaCl, 1 mM EDTA, pH 7.0. ApoHb was finally concentrated to 1.1 mM trough diafiltration in an Amicon^®^ stirred cell (Merck) with a 10 kDa cut-off membrane. Holo-α and β chains were equilibrated in CO and separately mixed at the final concentration of 312.5 µM with 625 µM apoHb (1:2 monomer molar ratio) in 5 mL of CO-equilibrated 100 mM potassium phosphate, 150 mM NaCl, 1 mM EDTA, pH 7.0. Solutions were incubated statically for about 65 hours at 4 °C, centrifuged for 10 minutes at 10.000 x g at 4 °C and loaded on a HiLoad 16/600 Superdex 75 prep grade size-exclusion column (GE Healthcare) connected to an ÄKTA Prime FPLC system (GE Healthcare), equilibrated with 100 mM potassium phosphate, 150 mM NaCl, 1 mM EDTA, pH 7.0. The peaks corresponding to the dimeric semiHbs were collected, aliquoted and stored at −80 °C until further use.

#### Spectroscopy and stoichiometry determination

Heme extraction experiments were carried out in the UV-vis range using a Cary 4000 spectrophotometer (Varian). Spectra were collected in the 350–700 nm range, while the heme-transfer rates were evaluated at 406 nm. The temperature of the cell holder was maintained at 20 °C using a circulating water bath, and experimental buffer was composed of 100 mM Tris/HCl, 150 mM NaCl, and 1 mM EDTA, pH 8.0. To measure the heme extraction, 1.5 µM hemoglobin (heme concentration) and 7.5 µM IsdB were rapidly mixed in a double-chamber cuvette (each chamber has 4.375 mm pathlength). The two chambers are separately filled with Hb and IsdB solutions and a spectrum is acquired that is the sum of the two proteins spectra. The solutions are then mixed by inverting the cuvette, initiation the reaction. This set-up allows to remove any artefact due to sample dilution and protein contribution. The stoichiometric ratio of IsdB:MetHb complex was assessed by following the decrease of absorbance at 406 nm in the presence of 5 µM MetHb (heme concentration) and increasing concentrations of IsdB wt in the range of 0.3–40 µM. Extinction coefficients used for the determination of OxyHb and MetHb concentrations are 125,000 cm^−1^ M^−1^ at 415 nm and 130,000 cm^−1^ M^−1^ at 406 nm, respectively, at pH 8.0.

### Surface plasmon resonance

SPR measurements were performed at 25 °C by using a Biacore X100 instrument (GE Healthcare). The StrepMAB-Immo™ (Iba, Goettingen, Germany) was immobilized onto the CM5 sensor chip surface (GE Healthcare) by using the Amine Coupling kit (GE Healthcare) and the HBS-EP + running buffer (GE Healthcare), following the procedure reported in refs. ^50,51^. Briefly, after activation of CM5 sensor chip, a solution of StrepMAB-Immo™ (50 μg/mL) in immobilization buffer (10 mM sodium acetate pH 5.0, GE Healthcare) was fluxed over the reactive matrix at a flow rate of 10 μL/min for 7 minutes. About 17000 resonance units (RU) of StrepMAB-Immo™ were immobilized onto both Flow cell (Fc) 1 and Fc2. The remaining unreacted sites were blocked by fluxing 1 M ethanolamine-HCl pH 8.5 (GE Healthcare) for 7 min with a flow rate of 10 μL/min over both Fc1 and Fc2. After equilibrating the surface with PBS buffer and 0.005% P20 surfactant (GE Healthcare), the ligand, IsdB wt (5 µg/mL), or its mutant IsdB^Y440A^, produced as Strep-Tag fusion proteins, was captured onto the Fc2 up to reach a signal of about 1500 RU. SPR measurements were performed by using a single-cycle kinetics^50,52,53^. In particular, five sequential increasing concentrations of Hb (0.01–1 µM) in the reaction buffer were fluxed over the sensor chip surface for 160 s with a flow rate of 30 μL/min, followed by a 160 s dissociation with running buffer and a final dissociation of 400 s with the same buffer, without intermediate regeneration. A wizard template was used to automatically perform the analytical cycles and the entire analysis. The kinetic parameters were extracted from SPR data through the BiaEvaluation software 2.1 (GE Healthcare), using the reference surface Fc1 to correct for systematic noise and instrument drift. Three start up cycles using buffer were carried out to equilibrate the surface, followed by a zero concentration cycle of analyte in order to have a blank response usable for double reference substraction^54^. The SPR response as a function of time (called sensorgram), was globally fitted by using a 1:1 binding model, a two-state reaction model or a two-state reaction irreversible model. Goodness of the fits was evaluated by χ^2^ value and residual plots. To extract the kinetic parameters of the IsdB:OxyHb interaction, the Langmuir 1:1 binding model, which assumes a simple reversible bimolecular reaction between the ligand and the analyte, was applied^55,56^. The model was modified to take into account for the mass transport effect for which the analyte was driven towards the sensor chip surface (*A*_*surface*_) or back again to the bulk solution (*A*_*bulk*_) with the same mass transfer coefficient (*k*_*t*_)^57^. The analyte, reaching the sensor chip surface, binds to the ligand leading to the formation of the ligand–analyte complex (*LA*), characterized by the association rate constant (*k*_*on*_) and the dissociation rate constant (*k*_*off*_):

$${A}_{bulk}\underset{{k}_{t}}{\overset{{k}_{t}}{\rightleftarrows }}{A}_{surface}+L.\underset{{k}_{off}}{\overset{{k}_{on}}{\rightleftarrows }}L.A$$

Accordingly, the variation of A_surface_, L and LA concentrations with time can be described by the following set of differential equations^58^.

$$\frac{{{\rm{d}}[{\rm{A}}}_{{\rm{surface}}}]}{{\rm{dt}}}={{\rm{k}}}_{{\rm{t}}}([{{\rm{A}}}_{{\rm{bulk}}}]-[{{\rm{A}}}_{{\rm{surface}}}])-({{\rm{k}}}_{{\rm{on}}}[{\rm{L}}][{{\rm{A}}}_{{\rm{surface}}}]-{{\rm{k}}}_{{\rm{off}}}[{\rm{LA}}])$$

$$\frac{{\rm{d}}[{\rm{L}}]}{{\rm{dt}}}=-\,({{\rm{k}}}_{{\rm{on}}}[{\rm{L}}][{{\rm{A}}}_{{\rm{surface}}}]-{{\rm{k}}}_{{\rm{off}}}[{\rm{LA}}])$$

$$\frac{{\rm{d}}[{\rm{LA}}]}{{\rm{dt}}}=({{\rm{k}}}_{{\rm{on}}}[{\rm{L}}][{{\rm{A}}}_{{\rm{surface}}}]-{{\rm{k}}}_{{\rm{off}}}[{\rm{LA}}])$$

Data collected with MetHb were analyzed according to a two-state reaction model, modified assuming the second step as irreversible (k_off2_ = 0)

$${\rm{A}}+{\rm{L}}\underset{{{\rm{K}}}_{{\rm{off}}1}}{\overset{{{\rm{K}}}_{{\rm{on}}1}}{\rightleftharpoons }}{\rm{AL}}\mathop{\longrightarrow }\limits^{{{\rm{K}}}_{{\rm{on}}2}}{\rm{AL}}^{\ast}$$

The sensorgram of the IsdB^Y440A^-MetHb interaction was analyzed by using the two-state reaction model, which describes a 1:1 binding of analyte (A), MetHb, to the immobilized ligand (L), IsdB^Y440A^ forming the AL complex, followed by a conformational change stabilizing the complex (AL^*^)^59^. Successively, the complex can dissociate through the reverse of the conformational change (two-state reversible):

$${\rm{A}}+{\rm{L}}\underset{{{\rm{K}}}_{{\rm{off}}1}}{\overset{{{\rm{K}}}_{{\rm{on}}1}}{\rightleftharpoons }}{\rm{AL}}\underset{{{\rm{K}}}_{{\rm{off}}2}}{\overset{{{\rm{K}}}_{{\rm{on}}2}}{\rightleftharpoons }}{\rm{AL}}^{\ast}$$

#### Determination of the dissociation constant for the tetramer-dimer equilibrium of hemoglobin

The tetramer-dimer dissociation equilibrium of Hb was analyzed through size-exclusion chromatography on a HiLoad 16/600 Superdex 75 column (GE Healthcare) connected to an ÄKTA Prime FPLC system (GE Healthcare) equilibrated with reaction buffer. Hb samples in the oxy and met forms were injected on the column at different concentrations. The dilution factors after elution were calculated according to^60^.

### Molecular modelling

#### Structure modeling

The following systems were modelled and submitted to Molecular Dynamics simulations. The system nomenclature here reported is retained along the manuscript. The procedure for preparing the models is reported in Fig. 4.

#### αβMetHb

Dimeric ferric human aquomethemoglobin (R state; PDB code 3p5q^61^).

#### IsdB:αβMetHb

This model represents the initial complex formed by IsdB and MetHb upon recognition, and was built starting from the X-ray structure of IsdB bound to the α chain of dimeric Hb (PDB code 5vmm^13^). The 5vmm structure shows a transition state, in which the heme group is displaced towards IsdB and the secondary structure of Hb, in particular at the helix F level, is severely altered by the presence of the hemophore. Thus, the original Hb dimer was replaced by the 3p5q structure, not altered by the hemophore presence. Residues at the IsdB:αHb chain interface were properly modelled to avoid clashes.

#### IsdB:βαMetHb

Following the same protocol, the hemophore structure was retained from the 5vmm X-ray structure, while the Hb replaced with the 3p5q structure. β and α chains were inverted to simulate IsdB bound to the β chain of dimeric MetHb.

#### holoIsdB:apo-αβMetHb

This model should represent the final step of the IsdB: αβMetHb interaction, upon the heme transfer and before the complex detachment. Here, the heme is located in the NEAT2 domain of a holo IsdB, while the MetHb α chain is in the apo form. In the IsdB:αβMetHb complex the IsdB NEAT2 domain was replaced with the X-ray structure of a heme-bound NEAT2 (PDB code 3rtl^22^). The heme was removed by the MetHb α chain and the residues at the IsdB:Hb interface were properly modelled to avoid clashes.

#### IsdB:αβOxyHb

Again, the 5vmm complex was used as a template. The dimeric Hb was replaced by the dimeric oxygenated form of ferrous human Hb (PDB code 2dn1^62^). The complex interface was properly modelled.

#### IsdB^Y440A^:αβMetHb

Starting from 5vmm X-ray structure, the same protocol reported for *IsdB:αβMetHb* was followed, adding a mutation of residue Tyr440 → Ala in IsdB.

#### System set-up

Ionizable residues were assigned standard protonation states at physiological pH. All histidines were kept in the default tautomeric (Nε-H) state, with the exception of the proximal and distal histidine^63^. In MetHb, the proximal and distal histidine residues coordinating the hemic iron were both Nδ-H protomers, while in the oxygenated hemoglobin the proximal and distal histidines were simulated as Nδ-H and Nε-H protomers, respectively. The parameters for the water- or oxygen-coordinating heme groups were calculated and tested in previous works^64,65^. Before the MD production, each complex was embedded in a cubic box and solvated in TIP3P water molecules, with addition of Na^+^ and Cl^−^ as counterions for neutralizing the total charge. The LINCS algorithm was employed to fix bond length between hydrogens and heavy atoms at the equilibrium distance. Periodic boundary conditions (PBC) were set, long range electrostatic effects were adjusted with the Particle-Mesh Ewald (PME) method, a cut-off value of 11 Å was fixed for both electrostatic and van der Waals interactions. The box was built using the BiKi software suite (http://www.bikitech.com/)^66,67^.

#### MD simulations

The complexes underwent 1·10^6^ energy minimization cycles using the steepest descendant algorithm, then a slow equilibration (four steps in isothermal-isochoric (NVT) and two steps in the isothermal-isobaric canonical (NPT) ensembles, respectively) was performed for heating the system from 0 to 300 K, with a temperature ramp of 50 K every 1 ns. The Newton’s equation was integrated every 2 fs, collecting frames every 10 ps, rescaling velocities with the Berendsen thermostat to control the temperature and coupling the system in NPT to a Parrinello-Rahman barostat. Lastly, a short equilibration of 2 ns in the NPT ensemble at 300 K was carried out before the 1 μs-long simulation in NVT ensemble. A total amount of about 6 μs was simulated.

#### Essential Dynamics

Essential motions in the near-constraints subspace were sampled by means of Essential Dynamics (ED) - or Principal Component Analysis (PCA) - to highlight the modes that contribute mostly to the overall dynamicity of the complex. After a least square fit to remove roto-translations, the covariance matrix of atomic displacement was calculated and then diagonalized, the eigenvectors were sorted by their eigenvalues. Among the resulting ranked top 10 eigenvectors, the first and the second describe the largest structural variance of the system and were thus taken into account for reducing complex motions to the essential subspace. In most cases their behavior is quite similar and the first, explaining the highest variance percentage, was chosen for the following PCA analysis. For each simulation, extreme conformations of the trajectory were collected and projected along the first eigenvector. The ED of IsdB and Hb were carried out separately in all the systems, to investigate how IsdB affects the motion and, thus, the function of Hb, and vice versa (Figs. 7 and S5).

The cosine content (*c*_*i*_) of the first eigenvector was extracted from the covariance matrix to analyze the protein behavior along the first principal component, which explains the highest percentage contribution to the overall variance. The cosine content (*c*_*i*_) is a value ranging between 0 (no cosine) and 1 (perfect cosine), where a *c*_*i*_ value close to 1 describes a motion resembling random diffusion close to an equilibrium conformation, while a *c*_*i*_ close to 0 indicates a mode likely linked to a structural transition. It is generally considered that *c*_*i*_ is higher than 0.5 when the structure oscillates around an equilibrium state, while *c*_*i*_ < 0.5 indicates a non-harmonic movement usually related to a structural transition having a biological meaning^68^.

All MD simulations and analyses were carried out with GROMACS software 4.5.1 version^69^. Plots were generated with MatLab software (MathWorks, Inc.)^70^. Pictures were prepared using PyMOL v1.7.6.4.

## Supplementary information

Supplementary Information
