# Supplementary material for: Interaction of human hemoglobin and semi-hemoglobins with the Staphylococcus aureus hemophore IsdB: a kinetic and mechanistic insight
Source: Sci Rep. 2019 Dec 9;9:18629. doi: 10.1038/s41598-019-54970-w (PMC6901573; doi:10.1038/s41598-019-54970-w)
Supplement: Supplementary file 1 — Supplementary Information [file 41598_2019_54970_MOESM1_ESM.pdf]

Supplementary Information

Interaction of human hemoglobin and semi-hemoglobins with the *Staphylococcus aureus* hemophore IsdB: a kinetic and mechanistic insight

Eleonora Gianquinto[1+], Ilaria Moscetti[2+], Omar De Bei[3+], Barbara Campanini[3,4], Marialaura Marchetti[1,4], Javier F. Luque[5], Salvatore Cannistraro[2], Luca Ronda[4,6,7], Anna Rita Bizzarri[2]*, Francesca Spyrakis[1]*, Stefano Bettati[4,6,7]*

[1] Department of Drug Science and Technology, University of Turin, Turin, 10125, Italy; [2] Department of Environmental and Biological Sciences, University of Tuscia, Viterbo, 01100, Italy; [3] Department of Food and Drug, University of Parma, Parma, 43124, Italy; [4] Interdepartment Center Biopharmanet-TEC, University of Parma, Parma, 43124, Italy; [5] Department of Nutrition, Food Science and Gastronomy, Faculty of Pharmacy and Food Sciences, Institute of Biomedicina (IBUB) and Institute of Theoretical and Computational Chemistry (IQTCUB), University of Barcelona, Santa Coloma de Gramenet, 08921, Spain; [6] Department of Medicine and Surgery, University of Parma, Parma, 43126, Italy; [7] Institute of Biophysics, National Research Council, Pisa, 56124, Italy.

*Corresponding authors: stefano.bettati@unipr.it; francesca.spyrakis@unito.it; bizzarri@unitus.it

[+]These authors contributed equally to this work

Table of contents

Figure S1. Purification of wild type IsdB and IsdB^{Y440A}……………………………………….S2

Figure S2. Determination of OxyHb and MetHb dissociation equilibrium.…………………...S3

Figure S3. Size-exclusion cromatography of semi-hemoglobins.……………….……………S4

Figure S4. Root Mean Square Deviation along simulation time………………………………...S5

Figure S5. Projection of the trajectories in the essential space……………………………….S6

Figure S6. Projection of the first eigenvector on the IsdB structure…………………………...S7

Figure S7. Intrachain hydrogen bonds occupancy in Hb helix F……………….…………...S8

Table S1. Occupancy of the hydrogen bonds formed by the heme group along the trajectories…………………………………………………………………………………S9

Table S2. Eigenvalues and cumulative contribution to the overall variance…………………..S10

Table S3. Cosine content of the first eigenvector, for each MD simulation………………..…S12

Figure S1. Purification of wild type IsdB and IsdB^{Y440A}. A. SDS-PAGE of wild type IsdB; from left to right (stacking gel was physically removed before scanning): Precision Plus unstained marker (Biorad), non-induced culture, induced culture, insoluble fraction, soluble fraction, flow through, final purified protein. **B.** SDS-PAGE of IsdB^{Y440A}; from left to right (stacking gel was physically removed before scanning): Precision Plus unstained marker (Biorad), non-induced culture, induced culture, insoluble fraction, soluble fraction, flow through, final purified protein. **C.** Absorption spectra of wild type (green continuous line) and Y440A (orange dashed line) IsdB.

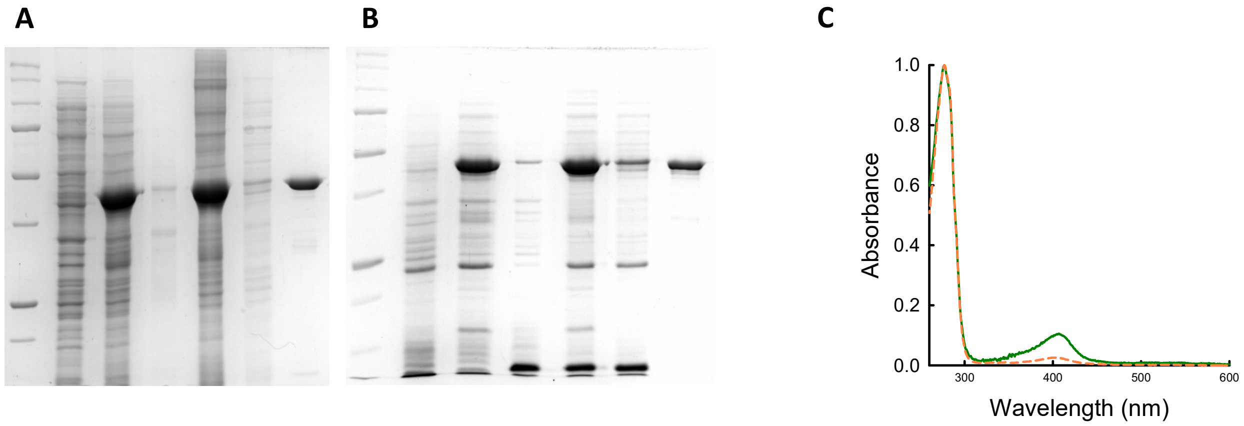

Figure S2. Determination of OxyHb and MetHb dissociation equilibrium. Dependence of OxyHb (open circles) and MetHb (closed circles) oligomeric state on globin concentration was determined by size-exclusion chromatography. Data were fitted to a hyperbolic function with an offset constrained to the dimer apparent molecular weight. Dashed and solid reference lines refer to the derived K_D for OxyHb (0.25 ± 0.05 μM) and MetHb (0.52 ± 0.06 μM), respectively.

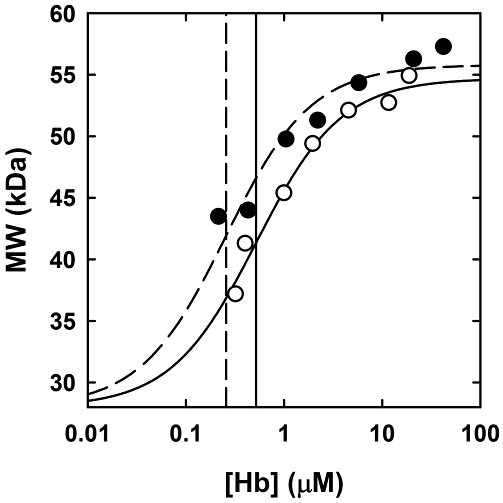

Figure S3. Size-exclusion cromatography of semi-hemoglobins. Semi-hemoglobins were purified on a HiLoad 16/600 Superdex 75 prep grade size exclusion column (GE Healthcare) equilibrated with 100 mM potassium phosphate, 150 mM NaCl, 1 mM EDTA, pH 7.0. **A**. A calibration curve was built by running myoglobin (17 kDa), carbonic anidrase (29 kDa), conalbumin (75 kDa) and blue dextran standards (GE Healthcare). Grey dashed dropped lines correspond to the expected molecular weights and retention times of hemoglobin tetramer, dimer and monomer. **B.** α- (dashed line) and β- (solid line) semiHb. The main peaks, corresponding to the semi-hemoglobins, were collected and aliquoted.

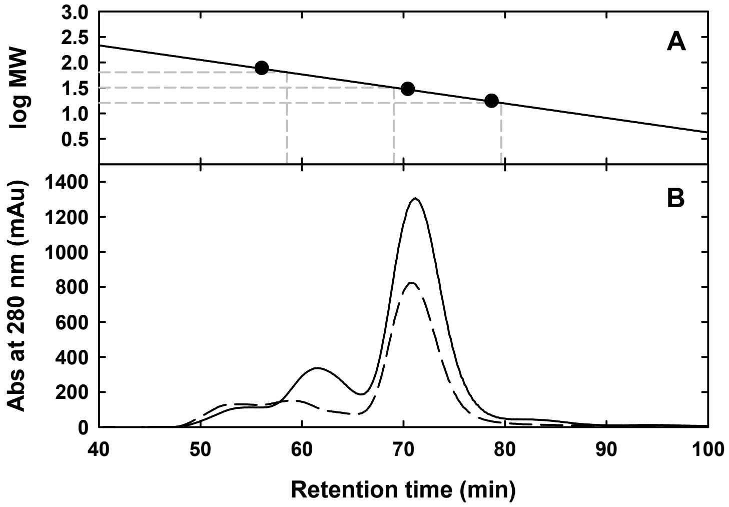

Figure S4. Root Mean Square Deviation along simulation time. Each graph reports the RMSD for the entire IsdB:Hb complex (red) and for the only backbone (black). **A.** αβMetHb. **B.** IsdB:αβMetHb. **C**. holoIsdB:apoαβMetHb. **D.** IsdB:αβOxyHb. **E.** $\text{IsdB}^{\text{Y440A}}$:αβMetHb. **F.** IsdB:βαMetHb.

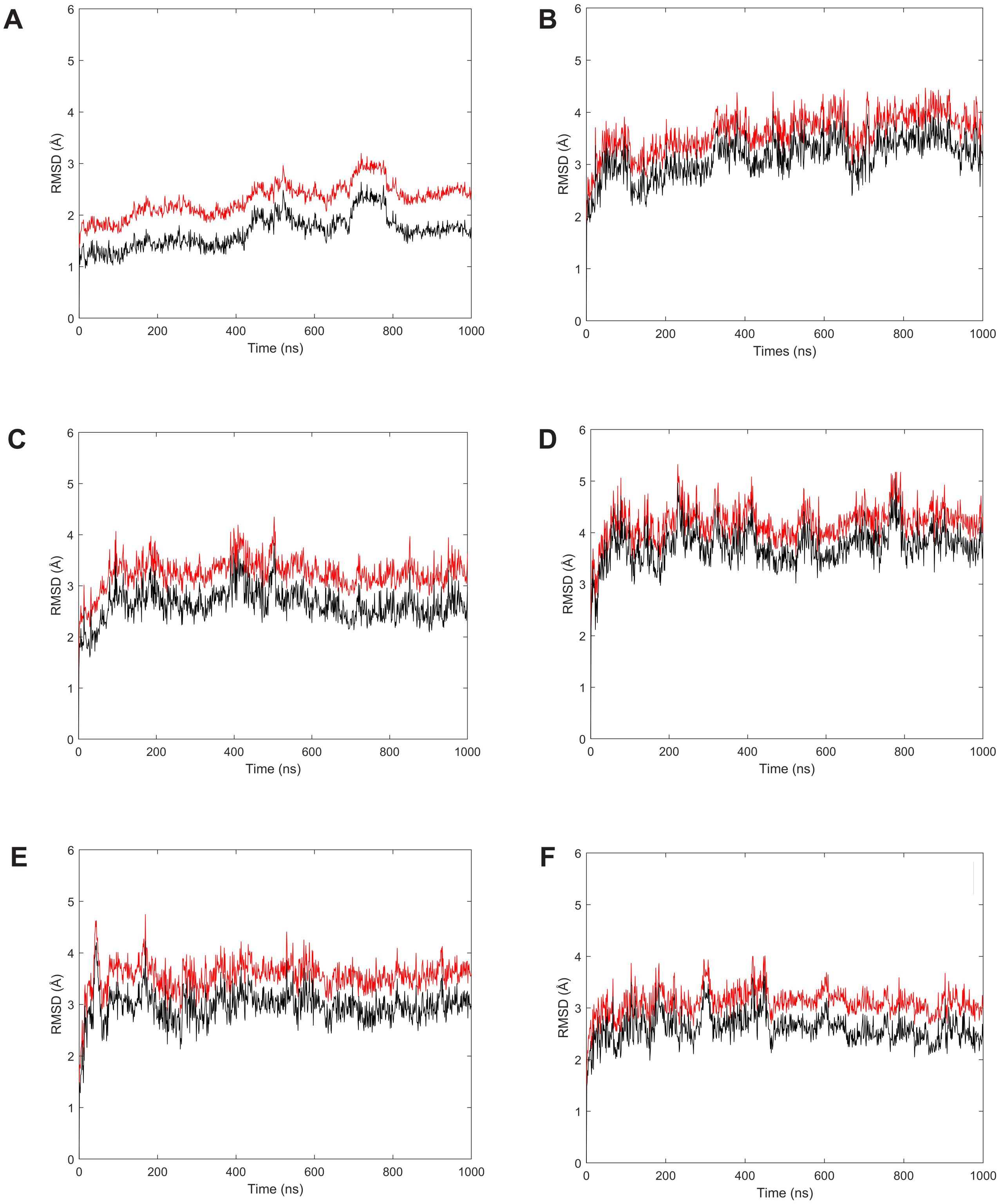

Figure S5. Projection of the trajectories in the essential space. Each frame of a trajectory is represented by a dot. The ED analysis, and the corresponding projection, has been performed separately for IsdB, Hb and the single Hb chains, as indicated in the graphs. **A.** αβMetHb. **B.** IsdB:αβMetHb. **C**. holoIsdB:apoαβMetHb. **D.** IsdB:αβOxyHb. **E.** IsdB^{Y440A}:αβMetHb. **F.** IsdB:βαMetHb.

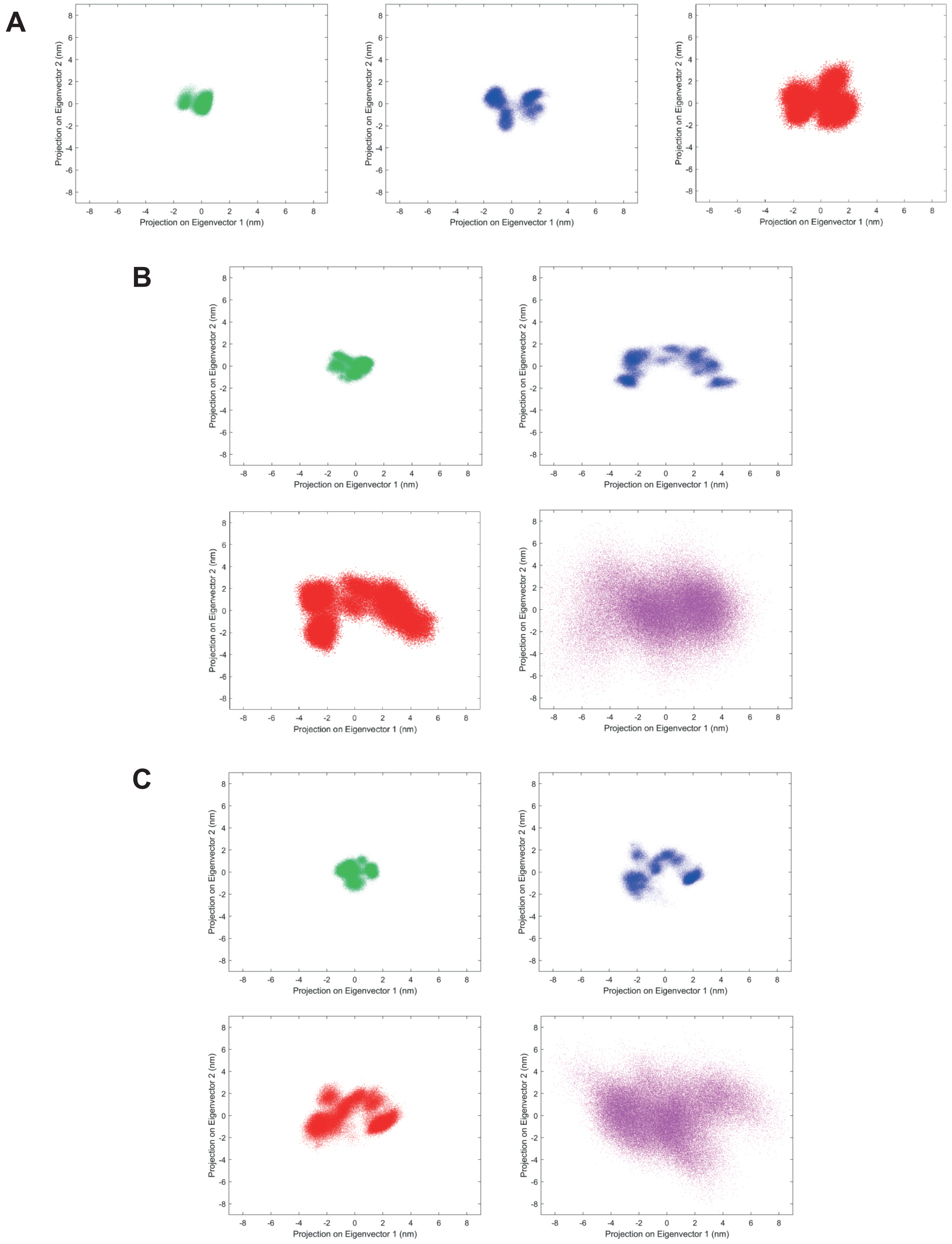

D

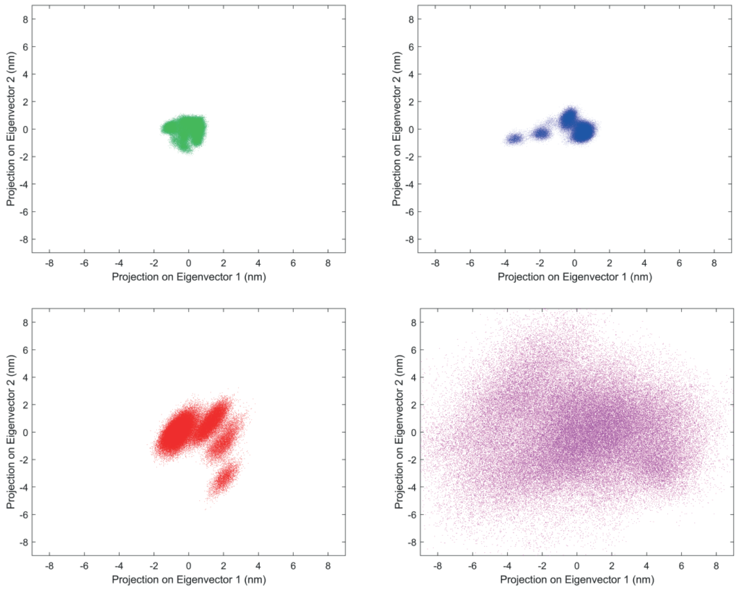
Projection on Eigenvector 1 (nm)
Projection on Eigenvector 2 (nm)

E

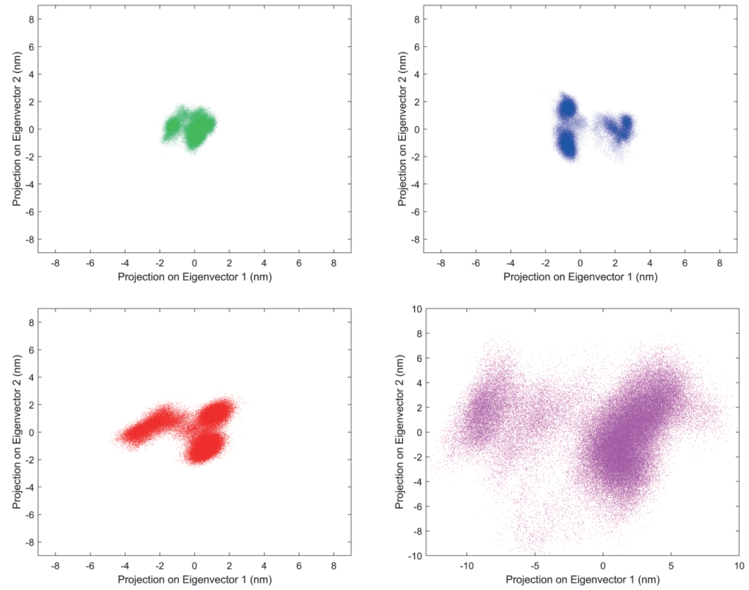
Projection on Eigenvector 1 (nm)
Projection on Eigenvector 2 (nm)

F

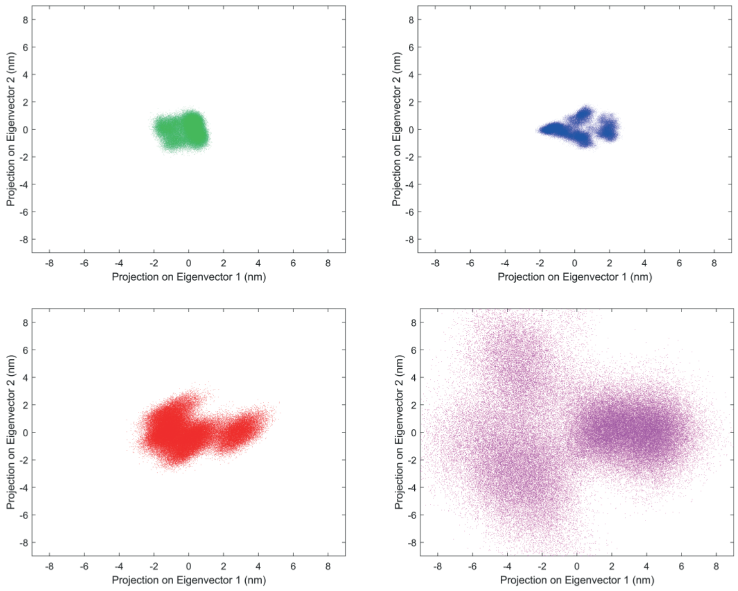
Projection on Eigenvector 1 (nm)
Projection on Eigenvector 2 (nm)

Figure S6. Projection of the first eigenvector on the IsdB structure (arrows, yellow tail and red head). IsdB:αβMetHb (**A**; pink), IsdB:βαMetHb (**B**; green), IsdB:αβOxyHb (**C**; yellow) holoIsdB:apoαβMetHb (**D**; pale cyan), and IsdB^{Y440A}:αβMetHb (**E**; grey).

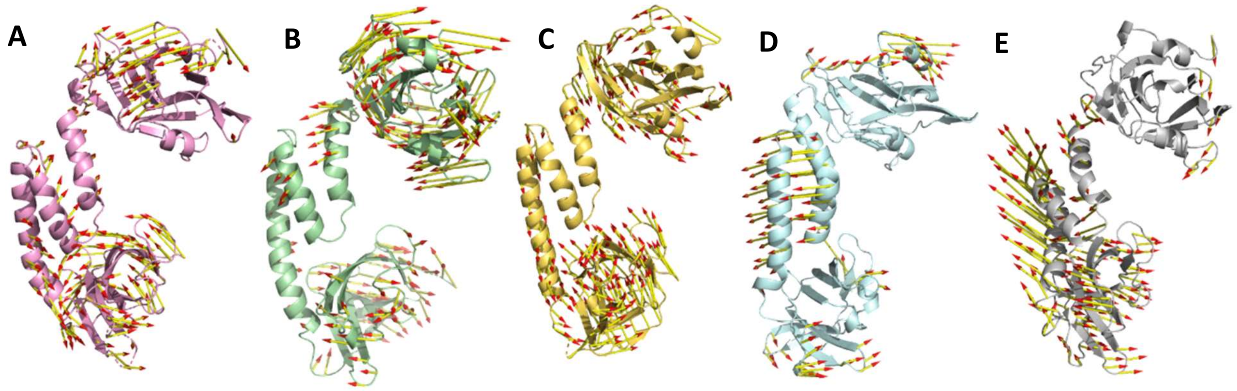

Figure S7. Intrachain hydrogen bonds occupancy in Hb helix F. A. Intrachain α F helix hydrogen bonds in αβMetHb (blue) are compared to those in α F helix IsdB:αβMetHb (light blue). **B.** Comparison between intrachain β F helix hydrogen bonds in αβMetHb (dark green) and those in β F helix IsdB:αβMetHb (light green). Occupancy value (%) is reported on the y axis, donor-acceptor pairs are on the x axis, with each bar indicating the occupancy of a donor-acceptor pair.

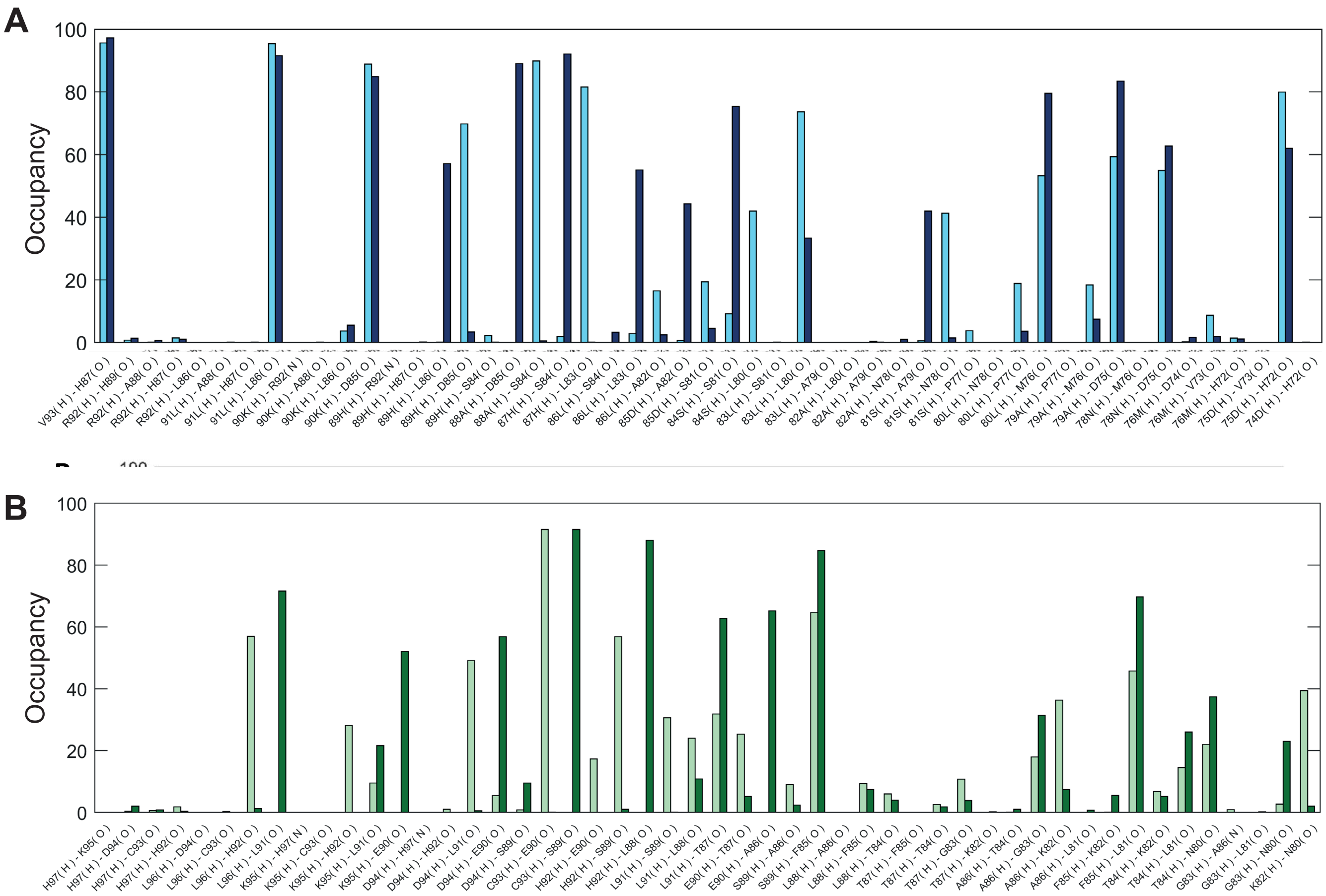

Table S1. Occupancy of the hydrogen bonds formed by the heme group along the trajectories. Only contacts with an occupancy higher than 20% are reported.

Donor-Acceptor pairs	IsdB:αβMetHb	holoIsdB:apoαβMetHb	IsdB:αβOxyHb	$IsdB^{Y440A}$:αβMetHb
$M362^{IsdB}$(H) - HEM(O2D)	-	47.4	-	-
$M362^{IsdB}$(H) - HEM(O1D)	-	50	-	-
$S361^{IsdB}$(HG) - HEM(O2D)	-	48.8	-	-
$S361^{IsdB}$(HG) - HEM(O1D)	-	45.3	-	-
$H58^{Hb}$(HD1) - HEM(O2A)	19.3	32.6	0.3	18.5
$H58^{Hb}$(HD1) - HEM(O1A)	18	34.5	0	20.1
$H45^{Hb}$(HE2) - HEM(O2A)	40.2	20.5	18.5	28.4
$H45^{Hb}$(HE2) - HEM(O1A)	38.3	20.2	17.1	25.9
$Y444^{IsdB}$(HH) - HEM(O2D)	38	-	16.9	42.2
$Y444^{IsdB}$(HH) - HEM(O1D)	38.6	-	15.6	42.1
$Y440^{IsdB}$(HH) - HEM(O2D)	29.7	-	7.8	-
$Y440^{IsdB}$(HH) - HEM(O1D)	28.2	-	4.9	-
$R290^{IsdB}$(H21) - HEM(O2D)	1.6	-	43.2	-
$R290^{IsdB}$(H21) - HEM(O1D)	1.6	-	48.2	-
$R290^{IsdB}$(H11) - HEM(O2D)	3.3	-	56.5	-
$R290^{IsdB}$(H11) - HEM(O1D)	3.2	-	52.2	-

Donor-Acceptor pairs	IsdB:βαMetHb
$H63^{Hb}$(HD1) - HEM(O2A)	16.3
$H63^{Hb}$(HD1) - HEM(O1A)	14.3
$Y444^{IsdB}$(HH) - HEM(O2D)	44.8
$Y444^{IsdB}$(HH) - HEM(O1D)	46.7
$Y440^{IsdB}$(HH) - HEM(O2D)	18.4
$Y440^{IsdB}$(HH) - HEM(O1D)	18.4

Table S2. Eigenvalues and cumulative contribution to the overall variance. After each MD simulation, eigenvalues were calculated and sorted in descending order for IsdB, for dimeric Hb and for each semiHb (Hbα and Hbβ chains), respectively. The tables below report the first 10 eigenvalues in nm^2 (left column), the cumulative variance (central column), and the percentage contribution of each eigenvalue to the overall variance.

αβMetHb								
Hbαchain			Hbβchain			Hbαβdimer		
e. val.	cum. var.	var. %	e. val.	cum. var.	var. %	e. val.	cum. var.	var. %
0,26	0,10	10,35	1,49	0,25	25,03	1,72	0,17	16,54
0,18	0,18	7,32	0,81	0,39	13,63	1,12	0,27	10,72
0,16	0,24	6,37	0,44	0,46	7,34	0,77	0,35	7,40
0,14	0,30	5,64	0,29	0,51	4,81	0,47	0,39	4,47
0,11	0,34	4,57	0,23	0,55	3,88	0,43	0,43	4,10
0,10	0,38	4,10	0,19	0,58	3,26	0,30	0,46	2,88
0,08	0,42	3,33	0,13	0,60	2,26	0,27	0,49	2,61
0,07	0,45	2,90	0,12	0,62	2,09	0,25	0,51	2,38
0,07	0,47	2,69	0,12	0,64	1,94	0,23	0,53	2,25
0,06	0,50	2,23	0,11	0,66	1,84	0,21	0,55	2,05

IsdB:αβMetHb											
Hbαchain			Hbβchain			Hbαβdimer			IsdB		
e. val.	cum. var.	var. %	e. val.	cum. var.	var. %	e. val.	cum. var.	var. %	e. val.	cum. var.	var. %
0,50	0,18	17,52	6,59	0,53	52,65	7,81	0,41	41,31	7,85	0,24	23,91
0,26	0,27	9,19	1,09	0,61	8,67	2,25	0,53	11,92	4,17	0,37	12,68
0,18	0,33	6,38	0,83	0,68	6,60	1,02	0,59	5,40	3,23	0,46	9,83
0,17	0,39	5,80	0,43	0,71	3,44	0,66	0,62	3,50	2,36	0,54	7,19
0,14	0,44	4,94	0,32	0,74	2,55	0,48	0,65	2,55	1,47	0,58	4,46
0,11	0,48	3,72	0,29	0,76	2,36	0,43	0,67	2,29	1,34	0,62	4,09
0,09	0,51	3,04	0,26	0,78	2,10	0,39	0,69	2,09	0,77	0,65	2,34
0,08	0,53	2,90	0,24	0,80	1,91	0,34	0,71	1,79	0,67	0,67	2,05
0,07	0,56	2,40	0,17	0,82	1,36	0,29	0,72	1,54	0,63	0,68	1,93
0,05	0,58	1,88	0,14	0,83	1,09	0,26	0,74	1,39	0,56	0,70	1,71

holoIsdB:apo-αβMetHb											
Hbαchain			Hbβchain			Hbαβdimer			IsdB		
e. val.	cum. var.	var. %	e. val.	cum. var.	var. %	e. val.	cum. var.	var. %	e. val.	cum. var.	var. %
0,58	0,17	17,01	2,95	0,23	23,37	3,52	0,23	23,37	7,58	0,23	23,11
0,34	0,27	10,05	1,06	0,31	8,11	1,22	0,31	8,11	4,35	0,36	13,27
0,22	0,33	6,38	0,67	0,38	6,97	1,05	0,38	6,97	3,09	0,46	9,41
0,18	0,39	5,31	0,52	0,44	5,56	0,84	0,44	5,56	2,52	0,53	7,67
0,14	0,43	4,00	0,44	0,49	5,15	0,78	0,49	5,15	1,84	0,59	5,59
0,11	0,46	3,27	0,33	0,52	3,33	0,50	0,52	3,33	1,25	0,63	3,80
0,11	0,49	3,15	0,28	0,55	2,84	0,43	0,55	2,84	0,99	0,66	3,02
0,09	0,52	2,63	0,25	0,58	2,59	0,39	0,58	2,59	0,77	0,68	2,34
0,09	0,54	2,50	0,20	0,60	2,10	0,32	0,60	2,10	0,60	0,70	1,84
0,07	0,56	2,16	0,19	0,62	2,04	0,31	0,62	2,04	0,52	0,72	1,59

IsdB:αβOxyHb											
Hbαchain			Hbβchain			Hbαβdimer			IsdB		
e. val.	cum. var.	var. %	e. val.	cum. var.	var. %	e. val.	cum. var.	var. %	e. val.	cum. var.	var. %
0,34	0,15	15,08	0,88	0,23	23,46	1,16	0,15	14,96	12,37	0,28	27,65
0,24	0,26	10,87	0,28	0,31	7,63	0,91	0,27	11,69	8,01	0,46	17,91
0,18	0,34	7,93	0,23	0,37	6,19	0,61	0,35	7,87	4,53	0,56	10,11
0,11	0,39	5,05	0,22	0,43	5,84	0,39	0,40	5,00	3,19	0,63	7,14
0,09	0,43	4,20	0,19	0,48	5,07	0,33	0,44	4,22	1,87	0,67	4,17
0,08	0,47	3,53	0,14	0,52	3,75	0,25	0,47	3,28	1,32	0,70	2,94
0,06	0,49	2,73	0,11	0,55	2,93	0,22	0,50	2,87	1,10	0,72	2,45
0,06	0,52	2,53	0,09	0,57	2,41	0,20	0,52	2,57	0,98	0,75	2,20
0,05	0,54	2,13	0,08	0,59	2,16	0,17	0,55	2,15	0,88	0,77	1,96
0,04	0,56	1,91	0,06	0,61	1,57	0,15	0,57	1,99	0,72	0,78	1,61

IsdBY440A:αβMetHb											
Hbαchain			Hbβchain			Hbαβdimer			IsdB		
e. val.	cum. var.	var. %	e. val.	cum. var.	var. %	e. val.	cum. var.	var. %	e. val.	cum. var.	var. %
0,36	0,13	12,72	1,76	0,26	25,64	2,34	0,22	21,78	18,26	0,34	33,73
0,27	0,22	9,41	1,37	0,46	19,89	1,30	0,34	12,05	7,19	0,47	13,28
0,16	0,28	5,83	0,47	0,52	6,79	0,69	0,40	6,36	5,54	0,57	10,24
0,14	0,33	5,09	0,30	0,57	4,40	0,54	0,45	5,04	3,38	0,63	6,24
0,12	0,37	4,27	0,21	0,60	3,11	0,37	0,49	3,47	2,40	0,68	4,43
0,11	0,41	3,98	0,16	0,62	2,38	0,27	0,51	2,48	1,57	0,71	2,90
0,11	0,45	3,81	0,15	0,64	2,21	0,23	0,53	2,10	1,35	0,73	2,50
0,08	0,48	2,98	0,13	0,66	1,88	0,20	0,55	1,90	1,13	0,75	2,09
0,07	0,50	2,37	0,11	0,68	1,63	0,19	0,57	1,76	0,91	0,77	1,67
0,06	0,53	2,18	0,11	0,70	1,57	0,18	0,59	1,65	0,79	0,79	1,47

IsdB:βαMetHb											
Hbαchain			Hbβchain			Hbαβdimer			IsdB		
e. val.	cum. var.	var. %	e. val.	cum. var.	var. %	e. val.	cum. var.	var. %	e. val.	cum. var.	var. %
0,43	0,13	12,59	1,40	0,30	30,16	2,56	0,24	23,95	14,12	0,30	29,73
0,32	0,22	9,19	0,30	0,37	6,54	0,83	0,32	7,75	9,74	0,50	20,50
0,22	0,28	6,37	0,27	0,42	5,74	0,63	0,38	5,92	4,04	0,59	8,51
0,20	0,34	5,96	0,25	0,48	5,32	0,43	0,42	4,02	1,94	0,63	4,09
0,17	0,39	4,90	0,19	0,52	4,19	0,36	0,45	3,37	1,57	0,66	3,32
0,15	0,43	4,41	0,17	0,56	3,74	0,29	0,48	2,70	1,41	0,69	2,97
0,14	0,48	4,13	0,14	0,59	3,04	0,28	0,50	2,58	1,03	0,71	2,17
0,09	0,50	2,66	0,10	0,61	2,26	0,26	0,53	2,46	0,94	0,73	1,98
0,08	0,53	2,33	0,09	0,63	2,03	0,22	0,55	2,02	0,88	0,75	1,85
0,08	0,55	2,23	0,08	0,65	1,81	0,21	0,57	1,92	0,83	0,77	1,76

Table S3. Cosine content of the first eigenvector, for each MD simulation.

	IsdB	Hb	Hbαchain	Hbβchain
MetHb	-	0.640431	0.104941	0.585568
IsdB:αβMetHb	0.6495	0.003	0.4318	0.0017
holoIsdB:apoαβMetHb	0.0784577	0.865061	0.565	0.839
IsdB:αβOxyHb	0.1432	0.5526	0.5735	0.401
$IsdB^{Y440A}$:αβMetHb	0.222721	0.610395	0.547	0.50987
IsdB:βαMetHb	0.753048	0.49547	0.09598	0.3933
